# A Potential Indicator Gene, *tetM*, to Assess Contamination by Antibiotic Resistance Genes in Greenhouses in South Korea

**DOI:** 10.1264/jsme2.ME24053

**Published:** 2024-12-28

**Authors:** Seunggyun Han, Raan Shin, Song-Hee Ryu, Tatsuya Unno, Hor-Gil Hur, Hanseob Shin

**Affiliations:** 1 School of Earth Sciences and Environmental Engineering, Gwangju Institute of Science and Technology (GIST), Gwangju, 61005, Republic of Korea; 2 Residual Agrochemical Assessment Division, National Institute of Agricultural Sciences, Wanju-gun, South Korea; 3 Department of Biological Sciences and Biotechnology, Chungbuk National University, Cheongju, Republic of Korea; 4 Center for Health Effects of Environmental Contamination, University of Iowa, W195 Chemistry Building, University of Iowa, Iowa city, Iowa, United States; 5 State Hygienic Laboratory, University of Iowa, Coralville, Iowa, United States

**Keywords:** antibiotic resistance gene, greenhouse, indicator, anthropogenic activity

## Abstract

Antibiotic resistance genes (ARGs) have been emerging as a concerning threat to both environment and public health. The continuous input of manure, irrigation water, and fertilizers increases the abundance of ARGs in agricultural environments. However, current risk assessments have focused on clinical settings, which are not applicable to environmental settings. Therefore, we herein aimed to identify and assess indicator genes to reduce the time and effort required for ARG surveillance. A nationwide ana­lysis of 322 ARGs and 58 mobile genetic elements (MGEs) was performed on 42 greenhouse and 19 control soil samples. The chemical properties and pH of soil were also investigated to characterize differences between greenhouse and control soil samples. The results obtained showed that the abundance of ARGS was significantly higher and ion concentrations were higher in greenhouse samples than in control samples. These results indicate that agricultural activities increased the abundance of ARGs. Furthermore, the abundance of core genes was significantly higher in greenhouse samples than in control samples, and the chemical characteristics of soil significantly differed between these samples. Among the discriminatory genes selected, *tetM* was identified as an ARG surveillance indicator gene based on its clinical relevance, prevalence in the soil resistome, and relationship with mobile genetic elements. The present results will contribute to the continuous and rapid surveillance of antibiotic resistance dissemination and proliferation in greenhouses in South Korea.

Antibiotic resistance genes (ARGs) have become one of the biggest emerging pollutants to both public and environmental health (Pruden *et al.*, 2006; Arias and Murray, 2009). Human pathogenic bacteria, such as *Bacteroidales*, *Escherichia coli*, *Salmonella*, and *Shigella*, acquire ARGs by horizontal gene transfer (HGT) due to their high sequence similarity to ARGs in environmental settings (Fischbach and Walsh, 2009; Forsberg *et al.*, 2012; Nesme *et al.*, 2014). Acquired resistance may curtail the efficiency of currently available antibiotics. Therefore, if effective antibiotics are not developed, approximately 11 million fatalities and a diminution of the economic size of the world by 0.1–3.1% are predicted to occur by 2050 (Fitchett and Atun, 2016). Another consequence is the reemergence of an era when antibiotics and treatments were limited in their ability to treat bacterial infections (Huddleston, 2014).

Terrestrial and aquatic environments, such as landfills, wastewater treatment plants (WWTP), river sediments, and soil, constitute a principal reservoir of ARGs and antibiotic-resistant bacteria (ARB) (Riesenfeld *et al.*, 2004; Shin *et al.*, 2021, 2022, 2023a). The soil environment contains 30% of noted ARGs as a significant environmental reservoir of ARGs (Byrne-Bailey *et al.*, 2009). Agricultural activities are regarded as a key supplier of selective pressure on soil for pollution by ARGs (Kuppusamy *et al.*, 2018). The amount of antibiotics used is approximately 4- to 5-fold higher in agricultural industries than in clinical settings, and 30–90% of antibiotics administered to livestock for disease prevention are excreted with manure (Zhu *et al.*, 2013; Caban *et al.*, 2018). Therefore, the application of manure to soil increases the abundance and diversity of ARGs and ARB (Heuer *et al.*, 2011; Udikovic-Kolic *et al.*, 2014), together with irrigation water from WWTP, heavy metals in animal feed, and airborne particles (Létourneau *et al.*, 2010; Li *et al.*, 2015). As a consequence, humans are exposed to the risk of ARGs and ARB transmission through the consumption of raw crops and meat produced from livestock (Sundin and Bender, 1996). Among various forms of agriculture, the closed system of a greenhouse with the continuous application of manure and fertilizers may result in persistent pollution by ARGs (Fang *et al.*, 2015; Wang *et al.*, 2020). A previous study reported that the amounts of antibiotic residues and ARGs were significantly higher in greenhouse soil than in uncovered field soil (Fang *et al.*, 2015). This difference may be attributed to several factors: the controlled environment of greenhouses often involves the intensive use of antibiotics for pest and disease control, leading to higher residues (Herencia *et al.*, 2011). Additionally, the lack of natural elements in greenhouses, such as rain and wind, may prevent the dispersion and degradation of these compounds, allowing them to accumulate over time (Vox *et al.*, 2010). The higher humidity and stable temperature conditions in greenhouses may also create a more favorable environment for the proliferation and persistence of ARGs (Herencia *et al.*, 2011). In South Korea, facility cultivation is conducted on approximately 81,195 ha (Jang *et al.*, 2020). Furthermore, the Korean government has propelled the installation of greenhouses for economic profit (Jang *et al.*, 2020). These endeavors increased the number of greenhouses by up to 1.7% every year from 1990 to 2018 according to the Ministry of Agriculture, Food and Rural Affairs of South Korea.

Current risk assessments have focused on clinical settings, which are not applicable to environmental settings (Berendonk *et al.*, 2015; Zhang *et al.*, 2021). “One-Health Perspective” refers to a comprehensive standpoint to cover the outlook of ARGs and ARB among humans, animals, and various environmental settings. Since the potential monitoring range of target ARGs is wide, difficulties are associated with simultaneously measuring all targets. The identification of proper candidate ARGs is needed (Tarek and Garner, 2023). Therefore, we consistently suggest candidate indicator genes to assess antibiotic resistance (AR) contamination in the agricultural environment. These reliable ARG indicators may be applied to rapid assessments with culture-independent methods (Berendonk *et al.*, 2015; Ishii, 2020). Relevant studies with indicator ARGs have been limited to aquatic environments (Narciso-da-Rocha *et al.*, 2014; Yang *et al.*, 2018), which has prompted the identification of indicator ARGs in the agricultural environment of South Korea. The selection of indicator genes will facilitate efficient assessments of AR contamination levels in the agricultural environment. Traditional methodologies for the quantification of antibiotic residues demand substantial labor and financial resources.

The present study hypothesized that 1) ARGs may be more abundant in greenhouse soil than in control soil, and 2) core ARGs in greenhouse soil may be selected as indicators. To establish a credible indicator of AR in greenhouse soil, we performed a nationwide ana­lysis of resistome and soil characteristics using 42 greenhouse soils and 19 control soils located in different provinces in South Korea. The results obtained herein will contribute to the establishment of a consistent AR surveillance system in the agricultural environment specifically for greenhouse soil.

## Materials and Methods

### Description of sampling sites and sample collection

**Fig. 1.**
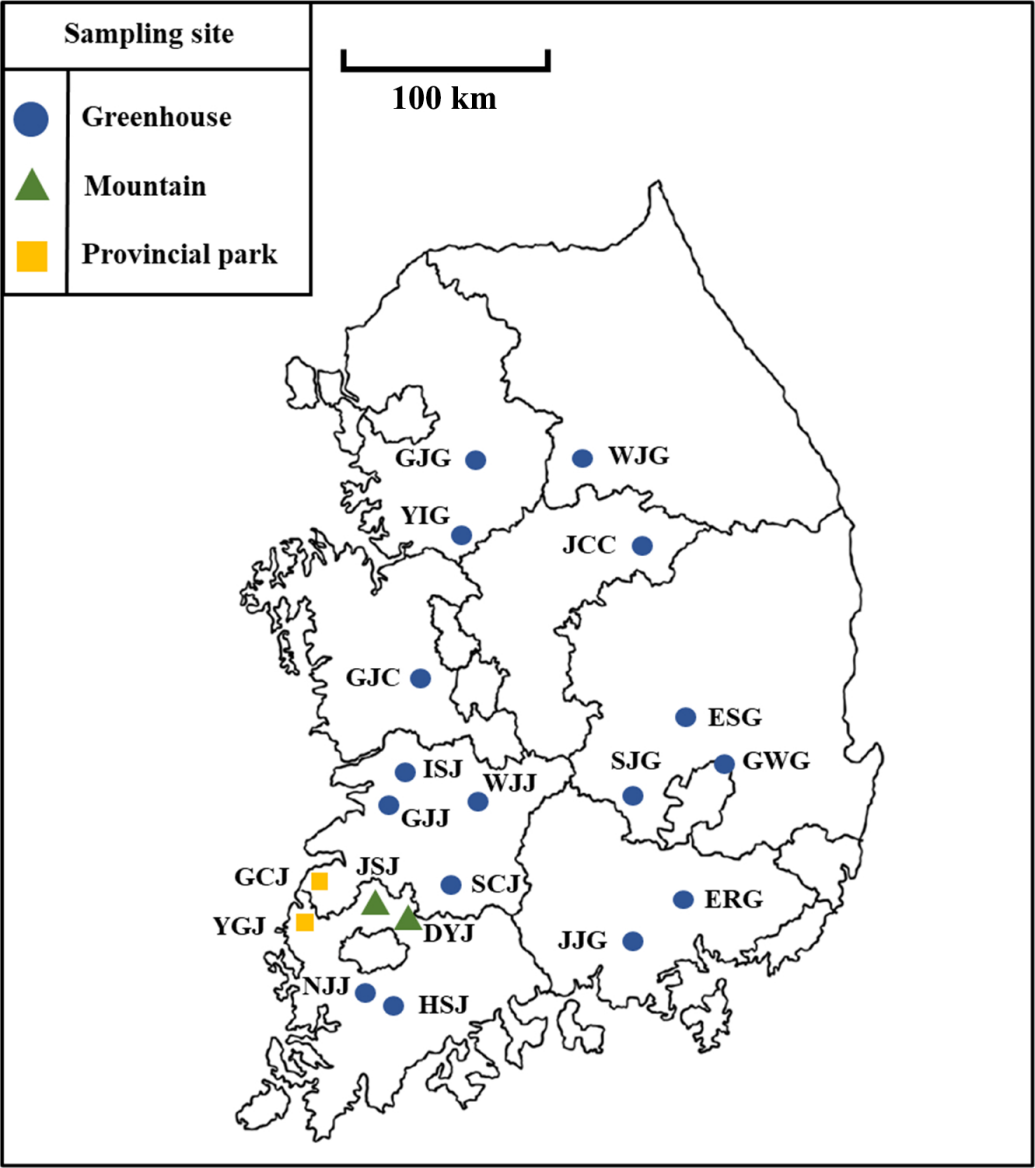
A map of South Korea showing 60 sampling sites (41 greenhouses, 11 mountain sites, and eight province parks). Blue, yellow, and green icons refer to the sites of greenhouses, provincial parks, and mountains, respectively. Mountain and provincial park samples were regarded as control samples. Each sample name was abbreviated based on the names of the city or mountain, ISJ: Iksan_Jeonbuk, SCJ: Sunchang_Jeonbuk, HSJ: Hwasun_Jeonnam, JCC: Jaecheon_Chungbuk, SJG: Seongju_Gyeongbuk, ESG: Euiseong_Gyeongbuk, GWG: Gunwi_Gyeongbuk, ERG: Euiryeong_Gyeongnam, JJG: Jinju_Gyeongnam, YIG: Yong-in_Gyeonggi, GCJ: Gochang_Jeonbuk, YGJ: Yeong-gwang_Jeonnam, GJC: Gongju_Chungnam, GJJ: Gimje_Jeonbuk, WJG: Wonju_Gangwon, WJJ: Wanju_Jeonbuk, GJG: Gwangju_Gyeongi.

Sites for sampling native greenhouse soils were selected based‍ ‍on the greenhouse location database of the Rural Development Administration (RDA) (https://www.nongsaro.go.kr/portal/portalMain.ps?menuId=PS00001). At least five greenhouses were selected from each of the eight provinces in South Korea for the nationwide ana­lysis (Fig. 1, Table S1 and 2). According to the RDA database, each of the vegetable cultivation crops had been cultivated for 5–30 years. The majority of samples were collected in September 2022 and 2023, except for nine samples collected from Jeonbuk and Jeonnam in September 2021 and March 2022. Each soil sample was collected from a depth range of 5 to 15‍ ‍cm and within a horizontal distance of 5 to 15‍ ‍cm from the crop. Eleven provincial park soil samples and 14 mountain soil samples were collected as controls from sites located at least 10 kilometers away from greenhouses to ensure the minimal impact of anthropogenic activities. The selection of control soil sites was based on land use history and the absence of recent anthropogenic challenges (*e.g.*, agricultural practices or chemical treatments). After their collection, soil samples were immediately placed in plastic bags, transferred to the laboratory under cool conditions, and stored at –20°C for further ana­lyses.

### Measurement of environmental parameters of soil samples

Soil chemical properties, including calcium (Ca^2+^), magnesium (Mg^2+^), ammonium (NH_4_^+^), phosphate (PO_4_^3–^), and nitrate (NO_3_^–^) concentrations and pH, were assessed. Eight grams of each soil sample was placed into a 50-mL conical tube with 40‍ ‍mL of sterilized distilled water. After an incubation for 1‍ ‍h in a shaking incubator at 30°C, the pH of the sample was detected using the Orion Star^TM^ A211 Benchtop pH Meter (Thermo Fisher). The portable ion analyzer, Rapid-d PIA-001 (Technell), was used to measure the concentrations of five ions (Ca^2+^, Mg^2+^, NH_4_^+^, PO_4_^3–^, and NO_3_^–^) according to the manufacturer’s instructions. Briefly, 200‍ ‍μL of the supernatant of the incubated mixture was transferred to each reagent provided by the company (Technell). After allowing the reaction to occur between the supernatant and reagent for 10‍ ‍min, ion concentrations in the solution were measured using the analyzer.

### DNA extraction

DNA was extracted from 0.5‍ ‍g of soil using the DNeasy^®^ PowerSoil^®^ kit (Qiagen) in compliance with the manufacturer’s instructions. The concentration of DNA was measured using the Nabi UV/Vis NANO Spectrophotometer (Microdigital).

### High-throughput quantitative PCR

The detection and quantification of 319 ARGs, 57 mobile genetic elements (MGEs), and 16S rDNA were conducted using the SmartChip Real-time PCR System (Wafergen Biosystems) (Chen *et al.*, 2016). The concentrations of all DNA extracts were adjusted to 20–30‍ ‍ng μL^–1^ with a total amount of 100‍ ‍μL through the Nabi UV/Vis NANO Spectrophotometer (Microdigital). A total of 319 ARGs belonging to 11 classes, 57 MGEs, and 16S rRNA gene primers were selected (Table S3) (Stedtfeld *et al.*, 2018). The volume of quantitative amplification was 100 nL and it contained 50 nL of 1× LightCycler 480 SYBR Green I Master Mix (Roche) (0.1‍ ‍mg mL^–1^), 20 nL of a DNA template (approximately 5‍ ‍ng‍ ‍μL^–1^), 500 nM of forward and reverse primers, and 19 nL of nuclease-free PCR-grade water. The Wafergen SmartChip Real-Time PCR Cycler loaded with the SmartChip was performed under the following protocol: initial denaturation at 95°C for 10‍ ‍min, followed by 40 cycles of denaturation at 95°C for 30‍ ‍s and annealing at 60°C for 30 s. The target gene copy number was calculated as follows: Copy number=10^(31–Ct)/(10/3)^, where Ct is the threshold cycle and the relative abundance of ARGs was divided by the copy number of 16S rDNA for ARG per bacterial cell (Looft *et al.*, 2012). A detection limit was set at a threshold cycle of 28 following the recommendations from Wafergen. The value of the threshold cycle was 31 and it was also used as the detection limit (Su *et al.*, 2015). Each sample was measured in triplicate. If all of the triplicates were not amplified, they were discarded (Zhang *et al.*, 2019). When the range of amplification efficiency was within 1.8–2.2, the Ct value was used in further ana­lyses (Looft *et al.*, 2012).

### Statistical ana­lysis

The total number of ARGs/MGEs and the relative abundance of ARGs were visualized by a boxplot with the R package “ggplot” (version 3.4.4) (Nearing *et al.*, 2022). The Shapiro test was conducted to verify the normality of samples. The significance of differences in soil properties between greenhouse and control soil samples was calculated using the Kruskal-Wallis test. Core genes were defined as ARGs and MGEs detected in >90% of samples. A heatmap for the relative abundance of core genes was generated using the R package “pheatmap” (version 1.0.12). Non-metric multidimensional scaling (NMDS) was performed on soil parameters using the Bray-Curtis dissimilarity metric, utilizing two axes to visualize data in the two-dimensional space. This ana­lysis was conducted using the R package “vegan” (version 2.6–4). Spearman’s correlation between soil components and core ARGs/MGEs was calculated with the installation of “corrplot”, “tidyverse”, and “colorspace” (Kabacoff, 2022). An assessment of the feature importance of core ARGs to examine their potential as genetic determinants of AR was conducted with random forest classification (RFC) (https://github.com/scikit-learn/scikit-learn?tab=readme-ov-file) based on the abundance of ARGs. Data were partitioned into two distinct data frames. The prediction process was iteratively performed with identifiers being randomly shuffled in each iteration. The average feature importance was computed, sorted, and the top 10 features were visualized in a bar chart. The decision-making tree model was constructed using the packages “party,” “caret,” tidyselect”, and “rpart” (Rhys, 2020).

## Results

### Physicochemical properties of greenhouse and control soils

**Fig. 2.**
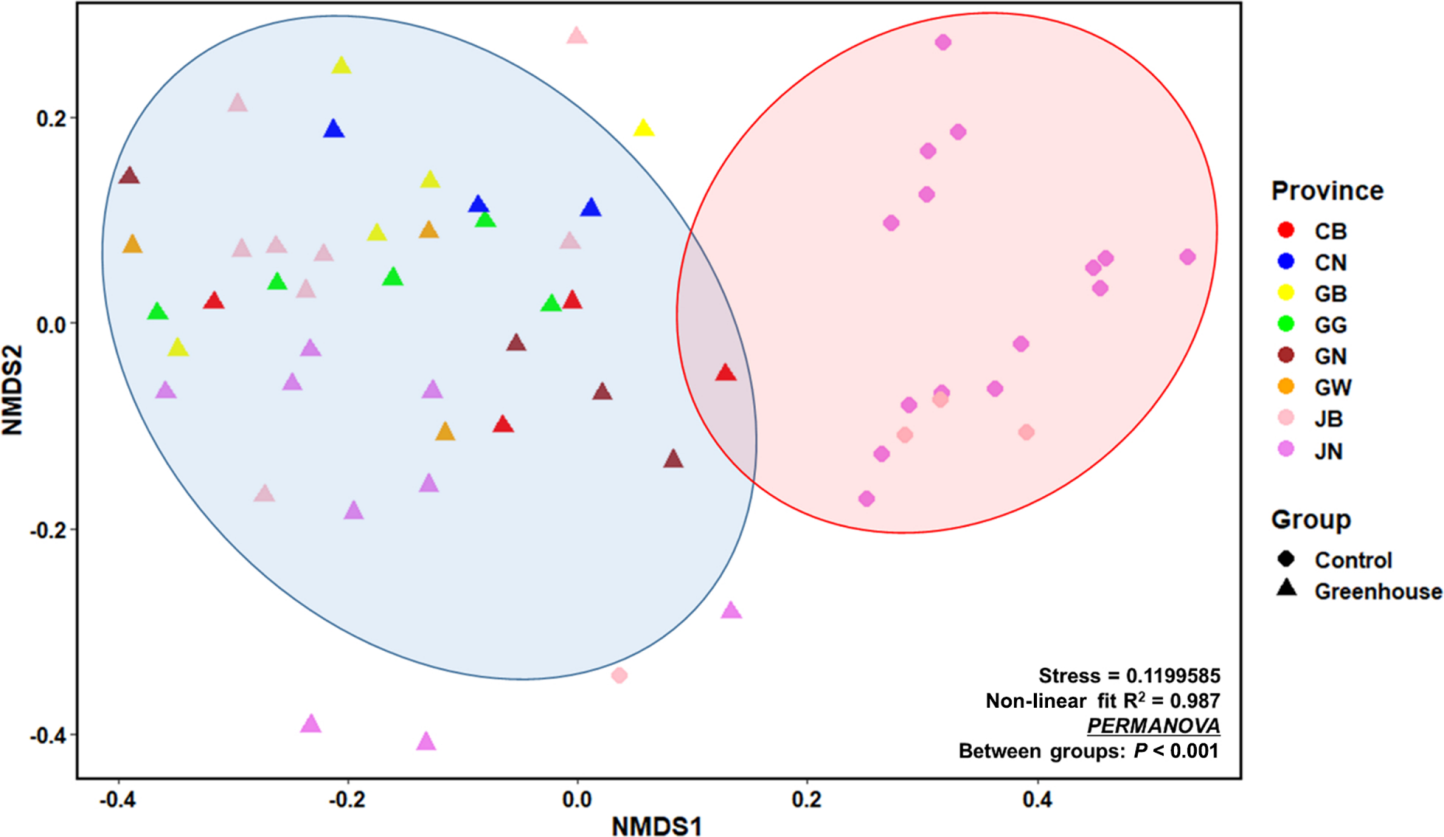
Two-dimensional NMDS using Bray-Curtis dissimilarity for soil components in greenhouse and control soil samples. Soil chemical properties were used as input data. The distance matrix was generated using the Bray-Curtis dissimilarity method. Colors indicate provinces regardless of shapes, and point shapes are a group of samples. Control soil samples are clustered within the red circle and greenhouse soil samples within the blue circle.

The concentrations of five ions (Ca^2+^, Mg^2+^, NH_4_^+^, NO_3_^–^, and PO_4_^3–^) and pH are shown in Table 1. Average pH in control and greenhouse samples were 5.4 and 6.0, respectively. All ion concentrations were higher in greenhouse samples than in control samples. NO_3_^–^, NH_4_^+^, Ca^2+^, Mg^2+^, and PO_4_^3–^ concentrations were 54-, 2-, 7-, 8-, and 4-fold higher, respectively, in greenhouse samples than in control samples. Two-dimensional NMDS using the Bray-Curtis dissimilarity of the soil properties of samples showed that greenhouse samples were more closely clustered together and significantly differed from control samples (Stress=0.987, Non-linear fit R^2^=0.987, *P*<0.001 between groups) (Fig. 2). Soil chemical parameters and pH were significantly higher in greenhouse samples than in control samples (Kruskal-Wallis test, *P*<0.05), while no significant differences were observed in the chemical characteristics of soil among the provinces exami­ned (the Kruskal-Wallis test, *P*=0.063).

### Abundance and diversity of ARGs and MGEs

**Fig. 3.**
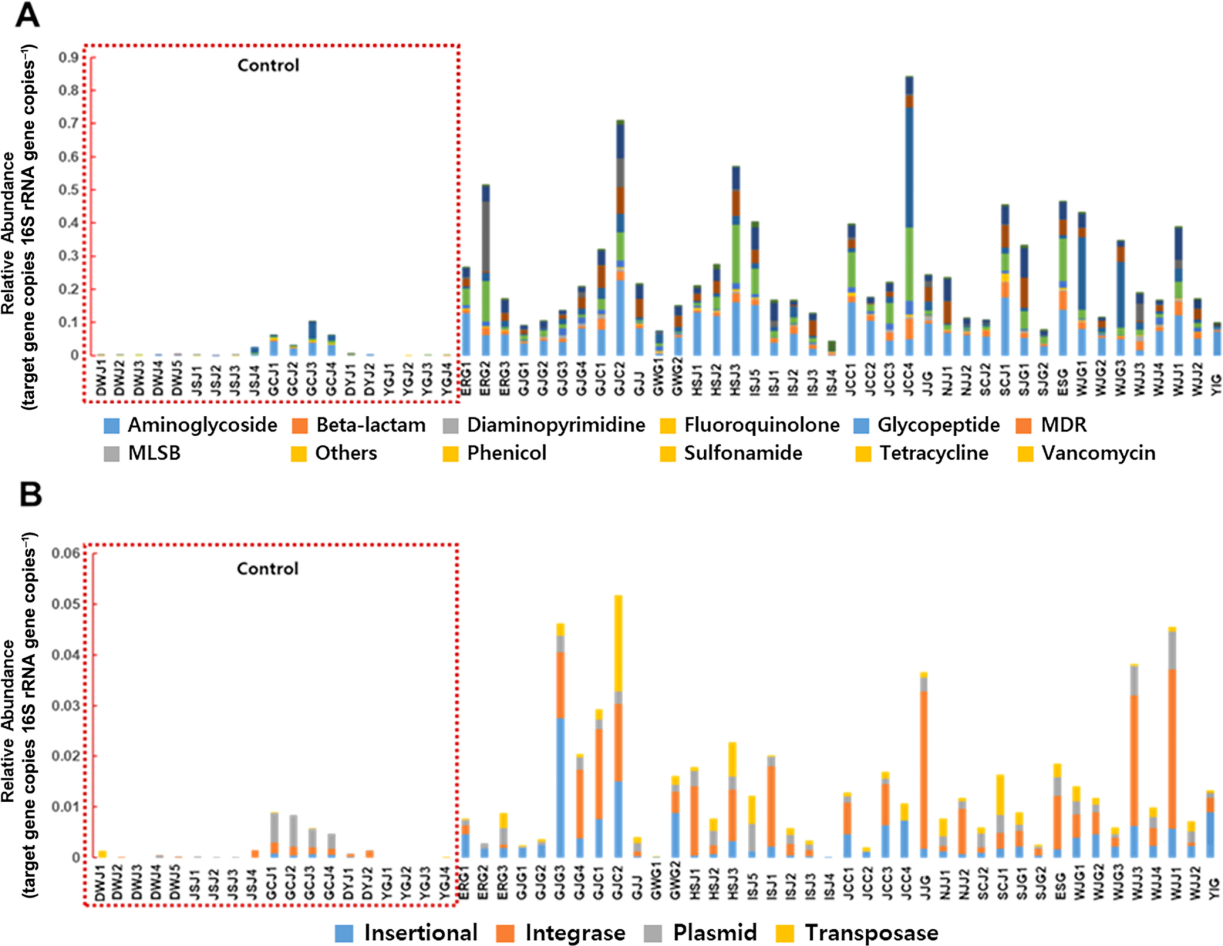
Relative abundance of ARGs and MGEs at each sampling site and group. The relative abundance of ARGs (A) and MGEs (B) was compared in each region. The relative abundance of ARGs and MGEs was then compared between control (*n*=19) and greenhouse soil (*n*=41) samples. The red dotted line in A and B clusters control soil samples.

**Fig. 4.**
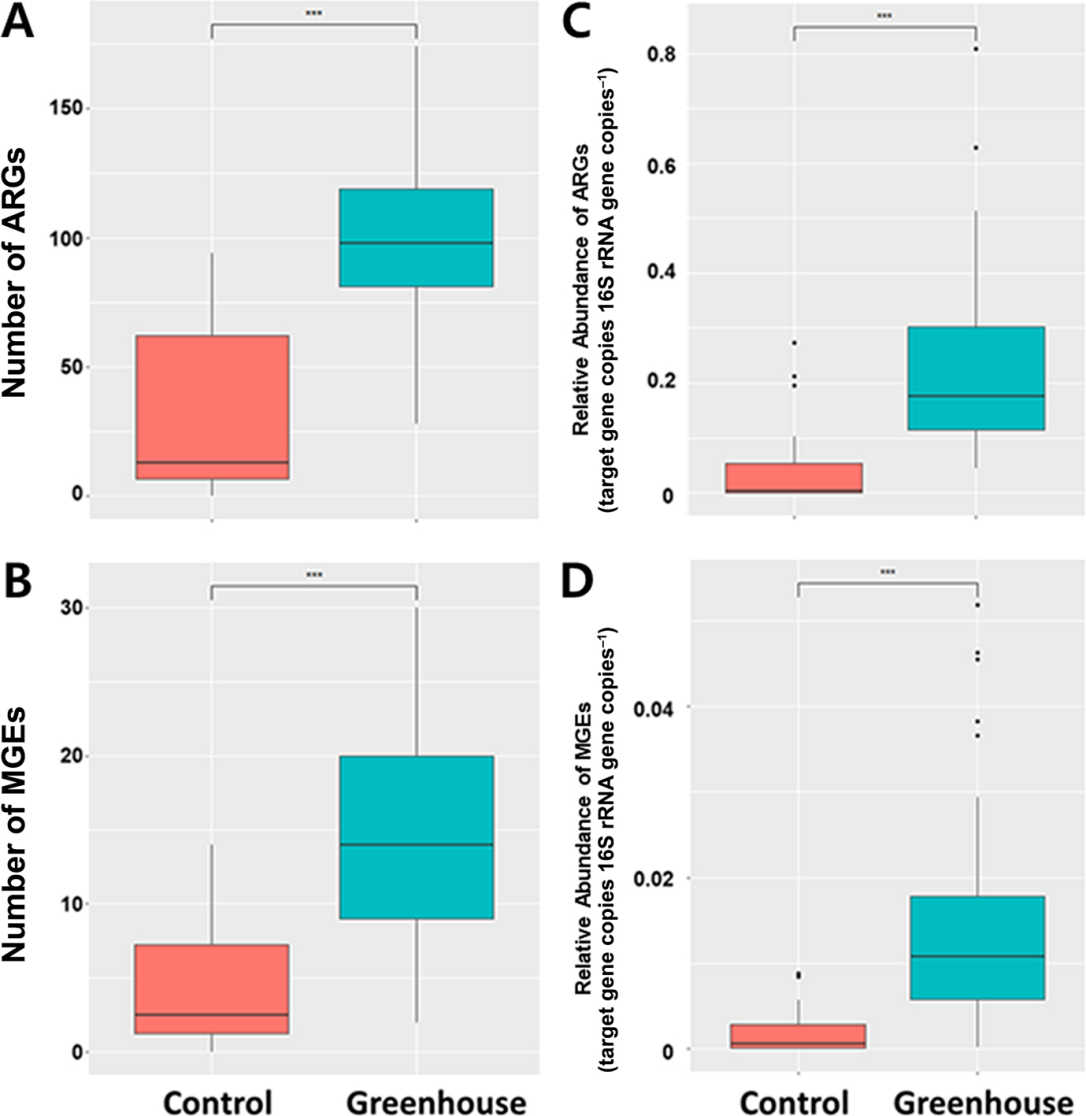
Number and relative abundance of total ARGs and MGEs at each sampling site and group. The red dotted line in a and b clusters control soil samples. Red and blue boxplots indicate control and greenhouse soils, respectively. The Kruskal-Wallis test was used to compare richness and relative abundance (target gene copies 16S rRNA gene copies^–1^). ***: *P*<0.001.

The relative abundance and diversity of ARGs in greenhouse and control samples are shown in Fig. 3. Control samples (mountain and provincial park samples) are in boxes with red dotted lines (Fig. 3A and B). ARGs and MGEs were rarely detected in control samples (DWJ, JSJ, GCJ, DYJ, and YGJ), but were more abundant at various concentrations in all greenhouse samples (Fig. 3A and B). In greenhouse samples, the highest abundance of ARGs was detected in JCC4, followed by GJC2 and ERG2, while the ISJ4 sample harbored the lowest abundance of ARGs. MGEs were dominant in GJC2, followed by GJG3 and WJJ1, but were rarely detected in ISJ4 and GWG1. Transposase-coding genes were the most abundant in GJC2, integrase-coding genes were frequently dominant in JJG, WJJ3, and WJJ1, and insertional sequences were the most frequently detected in GJG3. GJC2 had a greater abundance of ARGs and MGEs. Richness estimated by the number of ARGs and MGEs was significantly higher (*P*<0.001) in greenhouse samples than in control samples (Fig. 4A and B). The relative abundance of total ARGs and MGEs was also significantly higher (*P*<0.001) in greenhouse samples than in control samples (Fig. 4C and D).

### Discrimination of site types based on RFC and decision-making ana­lyses

**Fig. 5.**
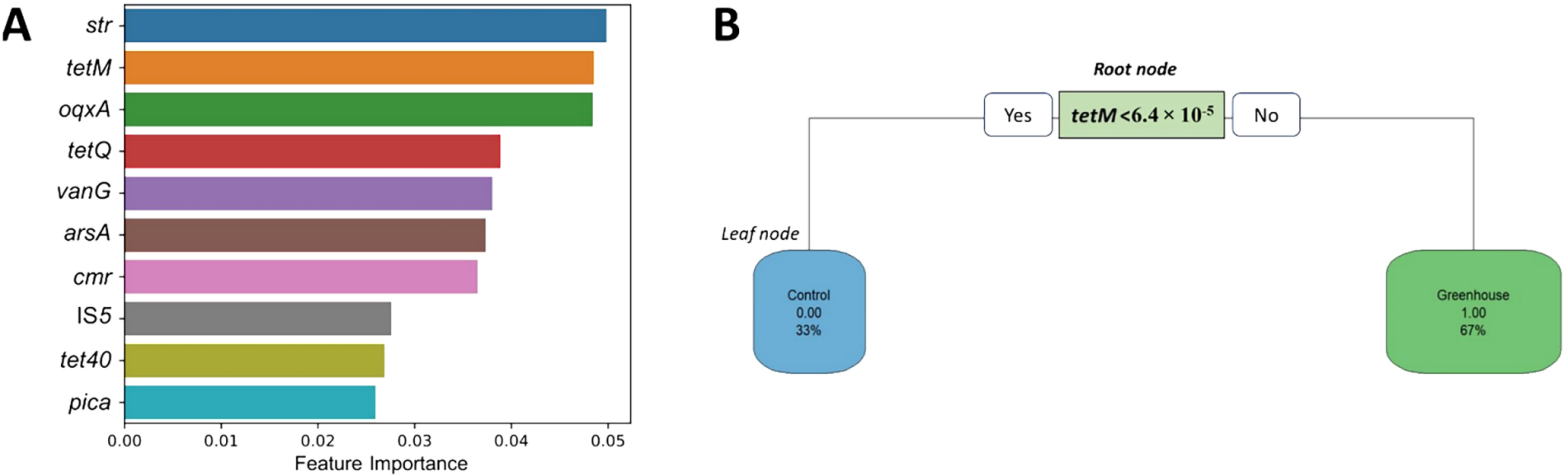
Random forest classification (A) and decision making (B) for the identification of significant indicator ARGs. The average feature importance was computed and sorted, and the top 10 features were visualized in a bar chart, providing insights into their significance in the model (A). ARGs were tested to discriminate soil types using a decision-making tree model (B).

The RFC ana­lysis ranked *str* (aminoglycoside resistance gene), *tetM* (tetracycline resistance gene), and *oqxA* (a gene encoding efflux pump) as the top three discriminatory genes between greenhouse and control soils, followed by *tetQ* (tetracycline resistance gene), *vanG* (glycopeptide resistance gene), *arsA* (heavy metal resistance gene), *cmr* (chloramphenicol resistance gene), IS*5* (insertional sequence), *tet40* (tetracycline resistance gene), and *pica* (MLSB resistance gene) based on their importance in discrimination (Fig. 5A). The discriminatory power of ARGs was estimated using a decision-making tree model. Among the top 10 ARGs, *tetM* was selected to construct the decision-making tree (Fig. 5B). Only two nodes were generated by the relative abundance of the *tetM* gene (either < or > 6.4×10^–5^ target gene copies 16S rRNA gene copies^–1^).

### Analysis of abundance and correlation of candidate indicator genes with soil characteristics

The relative abundance of 10 candidate indicator genes (*str*, *tetM*, *oqxA*, *tetQ*, *vanG*, *arsA*, *cmr*, IS*5*, *tet40*, and *pica*) was compared between greenhouse and control samples (Fig. S1). The abundance of eight candidate genes was significantly higher in greenhouse samples than in control samples (*P*<0.001). Greenhouse samples had various levels of ARGs. The abundance of genes, such as *tetM* (0–0.02 target gene copies 16S rRNA gene copies^–1^), *cmr* (0–0.03 target gene copies 16S rRNA gene copies^–1^), and *vanG* (0–0.18 target gene copies 16S rRNA gene copies^–1^), was higher in greenhouse samples than in control samples. In contrast, the abundance of ARGs was generally lower in control samples than in greenhouse samples. The relative abundance of the selected ARGs in most of the control samples was below the limit of quantification.

**Fig. 6.**
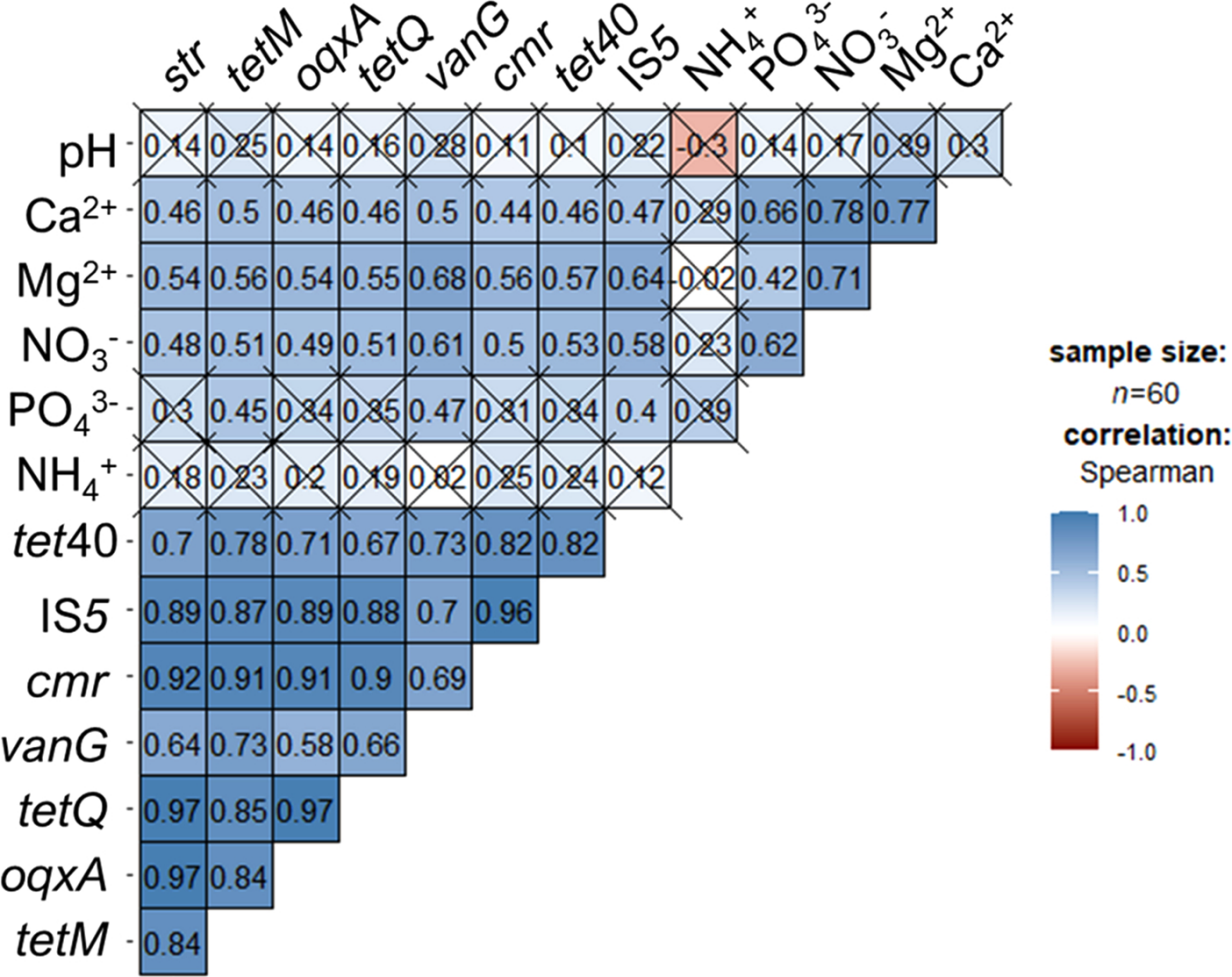
Relationships between candidate indicator genes and soil chemical components. Spearman’s correlation was applied as a non-parametric ana­lysis. The numbers and “×” in each box indicate the correlation coefficient and the lack of a correlation (*P*>0.05), respectively.

The relationships between eight candidate indicator genes and soil chemical components were exami­ned using a correlation ana­lysis (Fig. 6). Correlations were observed between some genes, such as *str*, *tetM*, *oqxA*, and *tetQ* (r=0.84–0.97, *P*<0.05). Additionally, correlations were found between ARGs and specific soil nutrients, including Ca^2+^, Mg^2+^, and NO_3_^–^ (r=0.44–0.68, *P*<0.05). PO_4_^3–^ also correlated with *tetM* (r=0.45, *P*<0.05), *vanG* (r=0.47, *P*<0.05), and IS*5* (r=0.40, *P*<0.05), but not with the other candidate indicator genes. No correlation was observed between NH_4_^+^ and candidate indicator genes (*P*>0.05).

## Discussion

### Impact of agricultural activities on the composition of greenhouse soil

Greenhouse cultivation has been developed to meet the growing demand for food production (Yuan *et al.*, 2022). However, this method frequently involves the input of manure, fertilizers, and irrigation water in limited areas, resulting in dynamic changes in the chemical components of‍ ‍soil (Yazgan *et al.*, 2008; Yuan *et al.*, 2022). The pres­ent‍ ‍study clearly showed that soil characteristics significantly differed between greenhouse and control samples in September 2022 and 2023, which is consistent with pre­vious findings (Berglund, 2015; Ouyang *et al.*, 2015). pH was higher in greenhouse samples than in control sam­ples,‍ ‍indicating potential changes in soil chemistry attribu­table to greenhouse agricultural activities. Furthermore, the ana­lysis of five key ions, Ca^2+^, Mg^2+^, NH_4_^+^, NO_3_^–^, and PO_4_^3–^,‍ ‍revealed differences between greenhouse and control samples, with significantly higher concentrations being observed in the former, particularly NO_3_^–^ (Herencia *et al.*, 2011). These results suggest that intensive and various agricultural activities, including the application of fertilizers, affect soil nutrient dynamics in the closed greenhouse system and promote the proliferation of ARGs and ARB (Fang *et al.*, 2015).

### Abundance of ARGs and MGEs in the agricultural environment

The assessment of ARG abundance in greenhouse and control soils represents a critical aspect of the present study, providing insights into the prevalence and distribution of ARGs in agricultural environments. The comparative ana­lysis revealed that the relative abundance of ARGs significantly varied between greenhouse and control samples, with the diversity and abundance of ARGs being higher in the former across multiple provinces in South Korea. These results are consistent with previous findings showing an increased abundance of ARGs in agricultural soils subjected to intensive farming practices (Heuer *et al.*, 2011; Marti *et al.*, 2013). It is important to note that local conventional practices to maintain the quality of soil in greenhouses involve the continuous replacement of soil with that outside the greenhouse. Therefore, indigenous greenhouse bacteria, which may be considered stable and competitive to thrive in local soils, acquire diverse ARGs through intensive agricultural activities in the greenhouse and are stable sources for the dissemination of ARGs through local practices for soil replacement in greenhouses (Han *et al.*, 2018). The application of manure has been reported to increase resistance genes to aminoglycoside, tetracycline, and sulfonamide in the agricultural environment (Cao *et al.*, 2020).

### Identification of candidate indicator genes

The need for an indicator gene for an AR contamination assessment is important for understanding the extent of ARG dissemination in various environments, particularly in‍ ‍agricultural soils. While conventional methods for measuring antibiotic concentrations, such as mass spectrometry, may not provide direct insights into the actual abundance of ARGs, the use of an indicator gene may be a practical solution for establishing a management policy on AR contamination in agricultural environments. The present results suggest that some ARGs, including core genes, act as‍ ‍candidate indicators in the agricultural environment, with‍ ‍a few overlapping with indicators in previous studies (Berendonk *et al.*, 2015; Zhang *et al.*, 2021). Four genes, *intI1*, *sul1*, *sul2*, and *tetM*, were previously proposed as AR indicator genes in manure-amended soil due to their low degradation rate and significant abundance (Wang *et al.*, 2018). Furthermore, seven genes, *intI1*, *intI2*, *ermB*, *ermC*, *qepA*, *qnrA*, and *qnrS*, were suggested as genetic probes for AR contamination (Wang *et al.*, 2019). We prioritized the necessity of identifying proper AR indicators in greenhouse facilities. The identification of indicator ARGs or MGEs needs to meet two requirements: i) clinical significance and ii) a relationship with MGEs for HGT (Berendonk *et al.*, 2015). Among the candidate indicator genes detected in the present study, the aminoglycoside resistance genes *str*, *strA*,* aadA5*, and *aac(6')-IIc* may be transmitted between clinical and environmental strains (Binh *et al.*, 2009; Jahan *et al.*, 2015) equipped in the genetic cassettes of *intl-1* encoding integrons (Partridge *et al.*, 2009; Jahan *et al.*, 2015). The genes *ermB*, *qnrS*, and *tetM* in this study satisfied the requirements of clinical relevance and transferability (Berendonk *et al.*, 2015). While they were selected as indicator genes, including *aadA5* for AR, in various environmental settings (Wang *et al.*, 2019; Zhang *et al.*, 2020; Shin *et al.*, 2023b), these genes were not identified as indicators in the agricultural environment by RFC and the decision-making model. In this study, *tetM* met the requirements of clinical relevance and a relationship with MGEs based on previous studies as well as its prevalence in the soil resistome in the present study. Therefore, *tetM* was selected as a discriminatory indicator ARG in greenhouses. In the future, we intend to investigate the relationship between the concentrations of applied and persistent agricultural antibiotics and the amount of *tetM* in agricultural soil in South Korea. The present study provides important insights for the identification of the potential indicator *tetM* gene, which may facilitate the effective management of AR contamination in agricultural environments.

### Relationship between candidate indicator genes and anthropogenic activity

#### Implications of candidate indicator genes in agricultural settings

Eight candidate indicator genes were selected, except for a heavy metal resistance gene (*arsA*) and efflux pump gene (*oqx A*). The identification of the agriculturally relevant ARGs, *str*, *tetD*, *tetM*, *tetQ*, and *tet40* implies that anthropogenic activities, including the application of oxytetracycline and streptomycin, has increased the abundance of specific ARGs. Streptomycin resistance genes, such as *str* and *strA*, are frequently found in agricultural settings due to the extensive use of streptomycin in both plant and animal agriculture for disease control (McManus *et al.*, 2002). In addition, the tetracycline resistance genes, *tet40*, *tetD*, *tetM*, and *tetQ* are prevalent in agricultural environments, which is largely due to the widespread use of tetracycline antibiotics in veterinary medicine and growth promoters in livestock production (Granados-Chinchilla and Rodríguez, 2017). These tetracycline resistance genes appear to be derived from anthropogenic sources (*e.g.*, manure application and the usage of antibiotics). Therefore, the presence of streptomycin and tetracycline resistance genes, particularly *tetM* as a powerful discriminatory indicator gene, may facilitate the selection and dissemination of resistance to human pathogens, which is of significant importance to public health.

#### Representatives of anthropogenic activities

The observed increases in soil nutrients, such as NH^4+^, NO^3–^, and PO_4_^3–^, due to anthropogenic activities, including fertilizer application, may contribute to the proliferation of ARGs in agricultural settings (Lu *et al.*, 2014; Tasho and Cho, 2016). Previous studies showed that high concentrations of these ions correlated with the increased abundance of ARGs, such as *ermB* and *tetM*, which was facilitated through mechanisms such as the conjugative transposon Tn916 under tetracycline exposure (Clewell *et al.*, 1995; Ali *et al.*, 2016). Agricultural practices involving the application manure and fertilizers may amplify specific ARGs, such as *tetM* in the present study. However, the results obtained herein do not conclusively establish the direct impact of anthropogenic activities on ARG proliferation.

### Limitations of the present study

It is important to note that information on irrigation and the application of manure or fertilizers was not available for this study. Due to the established impact of manure on the soil resistome (Caban *et al.*, 2018), the lack of data on manure application in the present study may limit our ability to fully understand the sources and drivers of ARGs in the soils exami­ned. Future research that includes detailed information on manure and fertilizer usage is needed to better elucidate their impact on the presence and abundance of ARGs in different agricultural settings.

## Conclusion

The present study elucidated the significant impact of agricultural activities on soil compositions and ARG abundance in greenhouse environments. Agricultural activities, including the use of antibiotics, application of manure, and irrigation, drive the proliferation of ARGs and MGEs in the agricultural environment. We identified the *tetM* gene as a potential indicator gene for assessing AR contamination in greenhouse soils based on its ubiquitous presence, transferability, relationship with nutrients driven by anthropogenic activities, and discriminatory power. The recognition of *tetM* as an indicator gene provides valuable insights for the consistent monitoring and management of AR contamination in agricultural ecosystems, which will mitigate the spread of ARGs and preserve environmental health. This study will contribute to advanced strategies for ongoing surveillance efforts to combat antibiotic resistance in agricultural settings.

## Citation

Han, S., Shin, R., Ryu, S.-H., Unno, T., Hur, H.-G., and Shin, H. (2024) A Potential Indicator Gene, *tetM*, to Assess Contamination by Antibiotic Resistance Genes in Greenhouses in South Korea. *Microbes Environ ***39**: ME24053.

https://doi.org/10.1264/jsme2.ME24053

## Supplementary Material

Supplementary Material

## Figures and Tables

**Table 1.** Components of greenhouse and control soils. The concentrations of ions (NH^4+^, PO_4_^3–^, NO^3–^, Mg^2+^, and Ca^2+^) in soil and pH were measured as soil components. The unit of each ion is ppm. Each result was expressed as the standard error with 95% confidence in the mean and rounded to two decimal places. The Kruskal-Wallis test was performed to compare the soil properties of greenhouse and control samples. (*: *P*<0.05, **: *P*<0.005, ***: *P*<0.001)

	NH_4_^+^ (ppm)	PO_4_^3–^ (ppm)	NO_3_^–^ (ppm)	Mg^2+^ (ppm)	Ca^2+^ (ppm)	pH
Greenhouse soils (*n*=41)	7.63±3.82*	84.87±44.61***	164.24±43.27***	16.41±3.34***	89.54±19.71***	5.97±0.24**
Control soils (*n*=19)	3.73±1.79	12.36±7.93	19.68±9.70	0.32±0.25	22.57±11.47	5.38±0.43
