# Supplementary material for: A Potential Indicator Gene, tetM, to Assess Contamination by Antibiotic Resistance Genes in Greenhouses in South Korea
Source: Microbes Environ. 2024 Dec 28;39(4):ME24053. doi: 10.1264/jsme2.ME24053 (PMC11821766; doi:10.1264/jsme2.ME24053)
Supplement: Supplementary file 1 — Supplementary Material [file 39_24053_s1.pdf]

**Table S1. Information of sampling sites associated with greenhouse soils located in Korea.** The information of crop cultivated in greenhouse was provided from Rural Development Administration(RDA).

| Sample | Province | City | GPS | Crop | Class of Crop | Years of Cultivation |
|---|---|---|---|---|---|---|
| **WJ_G1** | Gangwon | Weonju | 37°19'16"N 127°50'12"E | Red Peppers | Fruity Vegeatables | 15 |
| **WJ_G2** | Gangwon | Weonju | 37°18'13"N 127°59'11"E | Cucumber | Fruity Vegeatables | 15 |
| **WJ_G3** | Gangwon | Weonju | 37°16'40"N 127°49'34"E | Tomato | Fruity Vegeatables | 20 |
| **GG1** | Gyeonggi | Gwangju | 37°26'30"N 127°18'12"E | Tomato | Fruity Vegeatables | 15 |
| **GG2** | Gyeonggi | Gwangju | 37°26'06"N 127°18'43"E | Tomato | Fruity Vegeatables | 15 |
| **GG3** | Gyeonggi | Gwangju | 37°26'09"N 127°18'37"E | Mallow | Leafy Vegetables | 20 |
| **GG4** | Gyeonggi | Gwangju | 37°26'09"N 127°18'36"E | Mallow | Leafy Vegetables | 15 |
| **YI** | Gyeonggi | Yongin | 37°17'40"N 127°13'40"E | Mallow | Leafy Vegetables | 15 |
| **JJ** | Gyeongam | Jinju | 35°09'09"N 128°09'26"E | Red Peppers | Fruity Vegeatables | N/A |
| **ER1** | Gyeongam | Euiryeong | 35°28'39"N 128°20'32"E | Lettuce | Leafy Vegetables | N/A |
| **ER2** | Gyeongam | Euiryeong | 35°28'16"N 128°19'58"E | Lettuce | Leafy Vegetables | N/A |
| **ER3** | Gyeongam | Euiryeong | 35°28'19"N 128°20'04"E | Lettuce | Leafy Vegetables | N/A |
| **GW1** | Gyeongbuk | Gunwi | 36°15'33"N 128°33'02"E | Cherry Tomato | Fruity Vegeatables | N/A |
| **GW2** | Gyeongbuk | Gunwi | 36°15'22"N 128°32'43"E | Cherry Tomato | Fruity Vegeatables | N/A |
| **ES** | Gyeongbuk | Euiseong | 36°18'49"N 128°33'38"E | Cucumber | Fruity Vegeatables | N/A |
| **SJ1** | Gyeongbuk | Seongju | 35°51'39"N 128°20'53"E | Korean Melon | Fruity Vegeatables | N/A |
| **SJ2** | Gyeongbuk | Seongju | 35°50'04"N 128°20'19"E | Korean Melon | Fruity Vegeatables | N/A |
| **GJ_C1** | Chungnam | Gongju | 36°33'57"N 127°07'15"E | Red Peppers | Fruity Vegeatables | 24 |
| **GJ_C2** | Chungnam | Gongju | 36°32'52"N 127°07'12"E | Red Peppers | Fruity Vegeatables | 34 |
| **GJ_C3** | Chungnam | Gongju | 36°32'59"N 127°07'07"E | Red Peppers | Fruity Vegeatables | 28 |
| **GJ_J1** | Jeonbuk | Gimje | 35°50'39"N 126°47'15"E | Cherry Tomato | Fruity Vegeatables | 24 |
| **GJ_J2** | Jeonbuk | Gimje | 35°50'39"N 126°47'14"E | Cherry Tomato | Fruity Vegeatables | N/A |
| **JC1** | Chungbuk | Jecheon | 37°05'20"N 128°07'12"E | Red Peppers | Fruity Vegeatables | 19 |
| **JC2** | Chungbuk | Jecheon | 37°07'58"N 128°06'54"E | Napa Cabbage | Leafy Vegetables | 24 |
| **JC3** | Chungbuk | Jecheon | 37°08'42"N 128°13'31"E | Cucumber | Fruity Vegeatables | 20 |
| **JC4** | Chungbuk | Jecheon | 37°07'56"N 128°05'58"E | Spinach | Leafy Vegetables | 29 |
| **HS1** | Jeonnam | Hwasun | 35°00'22"N 126°56'32"E | Napa Cabbage | Leafy Vegetables | N/A |
| **HS2** | Jeonnam | Hwasun | 35°00'22"N 126°56'32"E | Napa Cabbage | Leafy Vegetables | 7 |
| **HS3** | Jeonnam | Hwasun | 35°00'49"N 126°55'46"E | Napa Cabbage | Leafy Vegetables | 7 |
| **NJ1** | Jeonnam | Naju | 35°03'11"N 126°47'52"E | Napa Cabbage | Leafy Vegetables | 28 |
| **NJ2** | Jeonnam | Naju | 35°03'11"N 126°47'52"E | Napa Cabbage | Leafy Vegetables | N/A |
| **SC1** | Jeonbuk | Sunchang | 35°23'44"N 127°12'13"E | Cherry Tomato | Fruity Vegeatables | 10 |
| **SC2** | Jeonbuk | Sunchang | 35°23'44"N 127°12'13"E | Lettuce | Leafy Vegetables | 10 |
| **WJ_J1** | Jeonbuk | Wanju | 35°51'47"N 127°10'51"E | Green Onion | Leafy Vegetables | 20 |
| **WJ_J2** | Jeonbuk | Wanju | 35°51'51"N 127°10'52"E | Green Onion | Leafy Vegetables | 20 |
| **WJ_J3** | Jeonbuk | Wanju | 35°51'47"N 127°11'04"E | Green Onion | Leafy Vegetables | N/A |
| **WJ_J4** | Jeonbuk | Wanju | 35°51'33"N 127°10'24"E | Green Onion | Leafy Vegetables | 20 |
| **IS1** | Jeonbuk | Iksan | 36°06'35"N 126°58'54"E | Cherry Tomato | Fruity Vegeatables | 21 |
| **IS2** | Jeonbuk | Iksan | 36°06'35"N 126°58'54"E | Cherry Tomato | Fruity Vegeatables | 20 |
| **IS3** | Jeonbuk | Iksan | 36°06'56"N 126°58'56"E | Cherry Tomato | Fruity Vegeatables | N/A |
| **IS4** | Jeonbuk | Iksan | 36°06'56"N 126°58'56"E | Tomato | Fruity Vegeatables | 5 |
| **IS5** | Jeonbuk | Iksan | 36°06'56"N 126°58'56"E | Tomato | Fruity Vegeatables | 5 |

***N/A: Not available**

**Table S2. Information of sampling sites associated with provincial park samples and mountain samples located in Korea.**

| Sample | Province | City | GPS |
|---|---|---|---|
| **GC1** | Jeonbuk | Kochang | 35°29'50"N 126°34'16"E |
| **GC2** | Jeonbuk | Kochang | 35°29'50"N 126°34'16"E |
| **GC3** | Jeonbuk | Kochang | 35°29'46"N 126°34'44"E |
| **GC4** | Jeonbuk | Kochang | 35°29'46"N 126°34'44"E |
| **YG1** | Jeonnam | Yeong-gwang | 35°11'57"N 126°33'03"E |
| **YG2** | Jeonnam | Yeong-gwang | 35°11'57"N 126°33'03"E |
| **YG3** | Jeonnam | Yeong-gwang | 35°11'30"N 126°32'41"E |
| **YG4** | Jeonnam | Yeong-gwang | 35°11'30"N 126°32'41"E |
| **BP1** | Jeonnam | Damyang | 35°18'50"N 126°52'31"E |
| **BP2** | Jeonnam | Damyang | 35°18'50"N 126°52'31"E |
| **BP3** | Jeonnam | Damyang | 35°18'46"N 126°52'28"E |
| **BP4** | Jeonnam | Jangseong | 35°20'04"N 126°52'20"E |
| **BP5** | Jeonnam | Jangseong | 35°20'03"N 126°52'22"E |
| **BT1** | Jeonnam | Jangseong | 35°16'54"N 126°50'30"E |
| **BT2** | Jeonnam | Jangseong | 35°17'02"N 126°50'30"E |
| **BT3** | Jeonnam | Jangseong | 35°16'50"N 126°50'31"E |
| **BT4** | Jeonnam | Jangseong | 35°17'37"N 126°50'21"E |
| **SI1** | Jeonnam | Damyang | 35°18'02"N 126°53'40"E |
| **SI2** | Jeonnam | Damyang | 35°18'02"N 126°53'40"E |

**Table S3. List of primers in this study**

| Gene | Classification | Forward primer | Reverse Primer |
|---|---|---|---|
| aac(3)-Ia | Aminoglycoside | ACGTTCTGCCAAAGTTTGAG | ACTGCCGGATCGTCAC |
| aac(3)-ib | Aminoglycoside | CAGCGAGACGTTCATCGC | CACGCTTCAGGTGGCTAATC |
| aac(3)-id_ie | Aminoglycoside | AGATAGTTATGCCCGCAACAAG | ACGCGCTGCGCCTATA |
| aac(3)-iid_iii_iif_iia_iie | Aminoglycoside | CGATGGTCGCGGTTGGTC | TCGGCGTAGTGCAATGCG |
| aac(3)-Via | Aminoglycoside | GTGTCCGTCGCCAAGGA | GGTGACGGCCTTGTCGA |
| aac(3)-xa | Aminoglycoside | GCAAGCGGTTCGTGACGTA | TCAGGTGCTCCTCGATCCAG |
| aac(6')I1 | Aminoglycoside | GGGAATTATCGGAATAGCTCTTGG | TTGGGCTGTTCTTCCTAGCTAA |
| aac(6')-Ib | Aminoglycoside | CGTCGCCGAGCAACTTG | CGGTACCTTGCCTCTCAAACC |
| aac(6')-Ie-APH(2")-Ia-2 | Aminoglycoside | CCAAGAGCAATAAGGGCATACCAA | GCCACACTATCATAACCACTACCG |
| aac(6')-ig | Aminoglycoside | GCGATGTTAGAAGCCTCAATTCG | CACACTTCGGCCTGTCGAA |
| AAC(6')-Iia | Aminoglycoside | CGACCCGACTCCGAACAA | GCACGAATCCTGCCTTCTCA |
| aac(6')-iic | Aminoglycoside | CAGTCTTTGGCTAATCCATCACAG | AACGAACCCGGCCTTCTC |
| aac(6')-ij | Aminoglycoside | ATGCCTGTATCTGAATCCCTGATG | GGCAATCGCTTGTTGAGTATCTG |
| aac(6')-im | Aminoglycoside | CGTGAGCATTATACAGAGCAATGG | CCATTTCCGTTCGTAGATATTGGC |
| aac(6')-ir | Aminoglycoside | GCTATAACGATCAGCAGCAAGC | CGCGATGCATGGCATGAC |
| AAC(6')-Is | Aminoglycoside | AAGCTTACTCTGGCCTGATCATG | TGCCTGAACGTCGATATTCAGG |
| AAC(6')-Iv | Aminoglycoside | TTGGCTTATACCGACACCCA | CCCGTTGCGATACCTGAAC |
| aac(6')-iw | Aminoglycoside | TGCGTCAGTTACTTACACGAAC | CCTGATGCATTGCATGACTGA |
| aac(6')-Iy | Aminoglycoside | GCCTCAATCCGCCACGATTA | ACGCGCTCTGTTTCCTCAAA |
| aac(6')-iz | Aminoglycoside | TGCGCCATGACTACGTGAAC | GACTGTCCGAAGCCAGTTCG |
| aacA43 | Aminoglycoside | CTTGGCCTACATTAGATTCAGCTC | GCTCTCAATCTTTGATAGGAGCAG |
| aacA-aphD | Aminoglycoside | AGAGCCTTGGGAAGATGAAGTTT | TTGATCCATACCATAGACTATCTCATCA |
| aacC2 | Aminoglycoside | ACGGCATTCTCGATTGCTTT | CCGAGCTTCACGTAAGCATTT |
| aacC4 | Aminoglycoside | CCAACACGACGCTGCATC | GCTGTCGCCACAATGTCG |
| aadA_99 | Aminoglycoside | GTTGTGCACGACGACATCATT | GGCTCGAAGATACCTGCAAGAA |
| aadA10 | Aminoglycoside | ACAGGCACTCAACGTCATCG | CGCGGAGAACTCTGCTTTGA |
| aadA16 | Aminoglycoside | ACGGTGGCCTGAAGCC | GAATTGCAGTTCCCGTCTGG |
| aadA17 | Aminoglycoside | TGTACGGCTCCGCAGTG | CACGGAATGATGTCGTCGTG |
| aadA21 | Aminoglycoside | ACGGCTCCGCAGTGGAT | GGCCACAGTAACCAACAAATCA |
| aadA22 | Aminoglycoside | CAATGACATTCTTGCGGGTATC | GACCTACCAAGGCAACGCTATG |
| aadA5 | Aminoglycoside | ATCACGATCTTGCGATTTTGCT | CTGCGGATGGGCCTAGAAG |
| aadA6 | Aminoglycoside | CCATCGAGCGTCATCTGGAA | CCCGTCTGGCCGGATAAC |
| aadA7 | Aminoglycoside | CACTCCGCGCCTTGGA | TGTGGCGGGCTCGAAG |
| aadA9 | Aminoglycoside | CGCGGCAAGCCTATCTTG | CAAATCAGCGACCGCAGACT |
| aadB | Aminoglycoside | CCTGCTTGGTGGGCAGAC | CGGCACGCAAGACCTCAA |
| aadD | Aminoglycoside | CCGACAACATTTCTACCATCCTT | ACCGAAGCGCTCGTCGTATA |
| aadE | Aminoglycoside | TACCTTATTGCCCTTGGAAGAGTTA | GGAACTATGTCCCTTTTAATTCTACAATCT |
| ant(4')-Ia | Aminoglycoside | GATGGCCGCTGACACATG | TCAACATTGCGCCATAGTGG |
| ant(6)-Ia | Aminoglycoside | TCGCCATGAGCTGCTGA | CCTATCATACTCCGGATAGGCATA |
| ant(6)-Ib | Aminoglycoside | AGAACATCCGACAGCACGTTC | CCAACCTTCCATGAAATCATTCGC |
| aph(2")-Id | Aminoglycoside | TGAGCAGTATCATAAGTTGAGTGAAAAG | GACAGAACAATCAATCTCTATGGAATG |
| aph(3")-ia | Aminoglycoside | TAACAGCGATCGCGTATTTCG | TCCGACTCGTCCAACATCAATA |
| aph(3')-Ib | Aminoglycoside | AACAGGTTTGGGAGGCGATG | CGCAACAAGCCTCTCCTGAA |
| APH(3')-VIa | Aminoglycoside | TCTCATGGCGATATCACGGATAG | TTTCCTCCGATGCATCCTCTC |
| aph(3')-viia | Aminoglycoside | CTCTCTCATGGAGATATGAGCGCTA | AATCCGGTTCAAGTCCCAACATG |
| aph(3')-viiia | Aminoglycoside | TCGGTATCCCGGTTGTGAG | ACACGAGGTACGGGAATCC |
| aph(4)-Ia | Aminoglycoside | CGCTCCCGATTCCGGAA | CACAGTTTGCCAGTGATACACA |
| APH(6)-Ia | Aminoglycoside | CGCTGGGAGCTGAAGAGG | AGCATCGTGCTGCTCTCC |
| aph3-III | Aminoglycoside | CAGAAGGCAATGTCATACCACTTG | GACAGCCGCTTAGCCGAA |
| aph4ib | Aminoglycoside | GGGAACACCGTGCTCACC | GTTGGTCCCGTGCAGGTC |
| aph6ic | Aminoglycoside | CACGACAACGTGCTCGAC | CCGTCTTCGGCGAACCA |
| aphA-1 | Aminoglycoside | TGAACAAGTCTGGAAAGAAATGCA | CCTATTAATTTCCCCTCGTCAAAAA |
| aphA3 | Aminoglycoside | AAAAGCCCGAAGAGGAACTTG | CATCTTTCACAAAGATGTTGCTGTCT |
| apmA | Aminoglycoside | GGCGCACATGCATTCATCA | CTATACTCCAGTCCCACCATTTGA |
| ArmA | Aminoglycoside | TCTTCGACGAATGAAAGAGTCG | GCTAATGGATTGAAGCCACAACC |
| spcN | Aminoglycoside | GCTATGTGCTGGTGGACTGG | GGAACCACTCGACGAACTCG |
| spec_aph | Aminoglycoside | GGTGCTGATATGAATGCCTTTGG | CATTGGGCGCATCAATAAATGG |
| str | Aminoglycoside | AATGAGTTTTGGAGTGTCTCAACGTA | AATCAAAACCCCTATTAAAGCCAAT |
| strA | Aminoglycoside | CCGGTGGCATTTGAGAAAAA | GTGGCTCAACCTGCGAAAAG |
| strB | Aminoglycoside | GCTCGGTCGTGAGAACAATCT | CAATTTCGGTCGCCTGGTAGT |
| ampC | Beta Lactam | CTGGCGCATACCTGGATTAC | GCCAGTTCAGCATCTCCCA |
| ampC/blaDHA | Beta Lactam | TGGCCGCAGCAGAAAGA | CCGTTTTATGCACCCAGGAA |
| beta_ccra | Beta Lactam | CACTGGCACGGCGATTGTA | CGGCAGCCAAACCACGATA |
| bl1acc | Beta Lactam | TGTTATCCGTGATTACCTGTCTGG | CTCAGCGAGCCAACTTCAAATA |
| bla1 | Beta Lactam | GCAAGTTGAAGCGAAAGAAAAGA | TACCAGTATCAATCGCATATACACCTAA |
| blaACC-1 | Beta Lactam | CACACAGCTGATGGCTTATCTAAAA | AATAAACGCGATGGGTTCCA |
| bla-ACT | Beta Lactam | AAGCCGCTCAAGCTGGA | GCCATATCCTGCACGTTGG |
| blaB | Beta Lactam | CGTGCCGGAGGTCTTGAATA | GGGATAGTAAACCTGAAACTCGGA |
| blaCARB | Beta Lactam | TGATTTGAGGGATACGACAACTCC | CTGTAATACTCCGAGCACCAA |
| blaCMY | Beta Lactam | AAAGCCTCATGGGTGCATAAA | ATAGCTTTTGTTTGCCAGCATCA |
| blaCTX-M-1_3_15 | Beta Lactam | CGTACCGAGCCGACGTTAA | CAACCCAGGAAGCAGGCA |
| blaFOXnew | Beta Lactam | CCTACGGCTATTCGAAGGAAGATAAG | CCGGATTGGCCTGGAAGC |
| blaGES | Beta Lactam | GCAATGTGCTCAACGTTCAAG | GTGCCTGAGTCAATTCTTTCAAAG |
| blaGOB | Beta Lactam | CTTGGGCTTGAATGCTCAGGTA | TGTATGGTCGTAGTGAGCCTGA |
| blaHERA | Beta Lactam | GGGCAACCGCATTCTGAC | GCATCTCCCACTTTATCGTCAC |

| | | | |
|---|---|---|---|
| blaIMI | Beta Lactam | ACATCTACACCTGCAGCAGTAG | AATCGCTTGGTACGCTAGCA |
| blaIMIR | Beta Lactam | AGCCGGACTAGAGCTTCATG | GGCAGAACTCATCATCTGCAAA |
| blaIND | Beta Lactam | CGCCTGTTAAACCCAACCTGTA | CGCTCTGTCATCATGAGAGTGG |
| bla-L1 | Beta Lactam | CACCGGGTTACCAGCTGAAG | GCGAAGCTGCGCTTGTAGTC |
| blaLEN | Beta Lactam | TGTTCGCCTGTGTGTTATCTCC | GCAGCACTTTAAAGGTGCTCAC |
| blaMIR | Beta Lactam | CGGTCTGCCGTTACAGGTG | AAAGACCCGCGTCGTCATG |
| blaMOX/blaCMY | Beta Lactam | CTATGTCAATGTGCCGAAGCA | GGCTTGTCCTCTTTCGAATAGC |
| blaOCH | Beta Lactam | GGCGACTTGCGCCGTAT | TTTTCTGCTCGGCCATGAG |
| blaOXA10 | Beta Lactam | CGACCGAGTATGTACCTGCTTC | TCAAGTCCAATACGACGAGCTA |
| blaOXY-1 | Beta Lactam | AAAGGTGACCGCATTCGC | CCAGCGTCAGCTTGCG |
| blaOXY-2 | Beta Lactam | CGTTCAGGCGGCAGGTT | GCCGCGATATAAGATTTGAGAATT |
| blaPAO/PDC | Beta Lactam | CGCCGTACAACCGGTGAT | GAAGTAATGCGGTTCTCCTTTCA |
| blaPER | Beta Lactam | GCAAATGAAGCGCAGATGC | GACCACAGTACCAGCTGGTA |
| blaROB | Beta Lactam | GCAAAGGCATGACGATTGC | CGCGCTGTTGTCGCTAAA |
| blaSFO | Beta Lactam | CCGCCGCCATCCAGTA | GGGCCGCCAAGATGCT |
| blaSHV-11 | Beta Lactam | TTGACCGCTGGGAAACGG | TCCGGTCTTATCGGCGATAAAC |
| bla-SME | Beta Lactam | GAGGAAGACTTTGATGGGAGGATTG | CGCTATATTGCAATGCAGCAGAAG |
| blaTEM | Beta Lactam | CGCCGCATACACTATTCTCAG | GCTTCATTCAGCTCCGGTTC |
| blaTLA | Beta Lactam | ACACTTTGCCATTGCTGTTTATGT | TGCAAATTTCGGCAATAATCTTT |
| blaVEB | Beta Lactam | CCCGATGCAAAGCGTTATG | GAAAGATTCCCTTTATCTATCTCAGACAA |
| blaVIM | Beta Lactam | GCACTTCTCGCGGAGATTG | CGACGGTGATGCGTACGTT |
| blaZ | Beta Lactam | GGAGATAAAGTAACAAATCCAGTTAGATATGA | TGCTTAATTTTCCATTTGCGATAAG |
| carB | Beta Lactam | GGAGTGAGGCTGACCGTAGAAG | ATCGGCGAAACGCACAAA |
| cefa_ampc | Beta Lactam | CAGGATCTGATGTGGGAGAACTA | TCGGGAACCATTTGTTGGC |
| cepA | Beta Lactam | AGTTGCGCAGAACAGTCCTCTT | TCGTATCTTGCCCGTCGATAAT |
| CfiA | Beta Lactam | GCAGCGTTGCTGGACACA | GTTCGGGATAAACGTGGTGACT |
| cfxA | Beta Lactam | TCATTCCTCGTTCAAGTTTTCAGA | TGCAGCACCAAGAGGAGATGT |
| cphA | Beta Lactam | GCGAGCTGCACAAGCTGAT | CGGCCCAGTCGCTCTTC |
| cphA2 | Beta Lactam | GTAACGCCTACTGGAAGTCCA | CAGCTTCTCCTTGAGAATGCAG |
| CTX-M | Beta Lactam | GCGATAACGTGGCGATGAAT | GTCGAGACGGAACGTTTCGT |
| imp-marko | Beta Lactam | GGAATAGAGTGGCTTAATTC | GGTTTAACAAAACAACCACC |
| KPC | Beta Lactam | GCCGCCAATTTGTTGCTGAA | GCCGGTCGTGTTTCCCTTT |
| mecA | Beta Lactam | GGTTACGGACAAGGTGAAATACTGAT | TGTCTTTTAATAAGTGAGGTGCGTTAATA |
| mecA-Staphylococci | Beta Lactam | CGCAACGTTCAATTTAATTTTGTTAA | TGGTCTTTCTGCATTCCTGGA |
| NDM new | Beta Lactam | GGCCACACCAGTGACAATATCA | CAGGCAGCCACCAAAAGC |
| nonmobile blaBEL | Beta Lactam | ATGTCCATGGCACAGACTGTG | CCTGTCTTGTCACCCGTTACC |
| nonmobile_blaADC | Beta Lactam | GGTATGGCTGTGGGTGTTATTCA | AGGCAAGGTTACCACTTGTATACG |
| pbp | Beta Lactam | CCGGTGCCATTGGTTTAGA | AAAATAGCCGCCCCAAGATT |
| Pbp5 | Beta Lactam | GGCGAACTTCTAATTAATCCTATCCA | CGCCGATGACATTCTTCTTATCTT |
| penA | Beta Lactam | AGACGGTAACGTATAACTTTTTGAAAGA | GCGTGTAGCCGGCAATG |
| PSE-1 | Beta Lactam | TTGTGACCTATTCCCCTGTAATAGAA | TGCGAAGCACGCATCATC |
| oqxA | Fluoroquinolone | GAGTCAACCTACCTCCACTATCA | GCTGCGAGTTATCCAGCAG |
| pmrA | Fluoroquinolone | TTTGCAGGTTTTGTTCCTAATGC | GCAGAGCCTGATTTCTCCTTTG |
| qnrA | Fluoroquinolone | AGGATTTCTCACGCCAGGATT | CCGCTTTCAATGAAACTGCAA |
| qnrB | Fluoroquinolone | GCGACGTTCAGTGGTTCAGA | GCTGCTCGCCAGTCGAA |
| QnrB4 | Fluoroquinolone | TCACCACCCGCACCTG | GGATATCTAAATCGCCCAGTTCC |
| qnrB46,47,48 | Fluoroquinolone | CGACGTTCAGTGGTTCAGATCTC | GCCAAGCCGCTCCATGAG |
| qnrD | Fluoroquinolone | CGCTGGAATGGCACTGTGA | GCTCTCCATCCAACTTCACTCC |
| QnrS1_S3_S5 | Fluoroquinolone | CCACTTTGATGTCGCAGATCTTC | CCCTCTCCATATTGGCATAGGAAA |
| qnrS2 | Fluoroquinolone | TCCCGAGCAAACTTTGCCAA | GGTGAGTCCCTATCCAGCGA |
| QnrVC1_VC3_VC6 | Fluoroquinolone | CTCACATCAGGACTTGCAAGAA | ATGAAGCATCTCGAAGATCAGC |
| QnrVC4_VC5_VC7 | Fluoroquinolone | TTCCTTTAAACGGGCAAACCTC | CGATACCTGATTCATGAAGCTAGC |
| vanA | Glycopeptide | GGGCTGTGAGGTCGGTTG | TTCAGTACAATGCGGCCGTTA |
| VanB | Glycopeptide | TTGTCGGCGAAGTGGATCA | AGCCTTTTTCCGGCTCGTT |
| vanC | Glycopeptide | CCTGCCACAATCGATCGTT | CGGCTTCATTCGGCTTGATA |
| vanC2_vanC3 | Glycopeptide | TGACTGTCGGTGCTTGTGA | GATAGAGCAGCTGAGCTTGTTC |
| vanD | Glycopeptide | CAGAGGAACATAATGTTTCGATAAAATCT | GCCGGATTTTGTGATTCCAA |
| vanG | Glycopeptide | TGTTTCGCAGAACCGTGTCAA | CCCTGCACTGTTCCATCTTCTC |
| vanHB | Glycopeptide | GAGGTTTCCGAGGCGACAA | CTCTCGGCGGCAGTCGTAT |
| vanHD | Glycopeptide | GTGGCCGATTATACCGTCATG | CGCAGGTCATTCAGGCAAT |
| vanRA | Glycopeptide | CCCTTACTCCCACCGAGTTTT | TTCGTCGCCCCATATCTCAT |
| vanRB | Glycopeptide | GCCCTGTCGGATGACGAA | TTACATAGTCGTCTGCCTCTGCAT |
| vanRC | Glycopeptide | TGCGGGAAAAACTGAACGA | CCCCCCATACGGTTTTGATTA |
| vanRC4 | Glycopeptide | AGTGCTTTGGCTTATCTCGAAAA | TCCGGCAGCATCACATCTAA |
| vanRD | Glycopeptide | TTATAATGGCAAGGATGCACTAAAGT | CGTCTACATCCGGAAGCATGA |
| vanSA | Glycopeptide | CGCGTCATGCTTTCAAAATTC | TCCGCAGAAAGCTCAATTTGTT |
| vanSB | Glycopeptide | GAAGATAAAGAGGGAAGCGTACTC | CCGAATTGTCAGCCCTTGATAA |
| vanSC | Glycopeptide | ATCAACTGCGGGAGAAAAGTCT | TCCGCTGTTCCGCTTCTT |
| vanTC | Glycopeptide | ACAGTTGCCGCTGGTGAAG | CGTGGCTGGTCGATCAAAA |
| vanTE | Glycopeptide | GTGGTGCCAAGGAAGTTGCT | CGTAGCCACCGCAAAAAAAT |
| vanTG | Glycopeptide | CGTGTAGCCGTTCCGTTCTT | CGGCATTACAGGTATATCTGGAAA |
| vanWB | Glycopeptide | CGGACAAAGATACCCCCTATAAAG | AAATAGTAAATTGCTCATCTGGCACAT |
| vanXA | Glycopeptide | TCGTTGGGACGCTAAATATGC | GGACGGTAACCGTCCCATA |
| vanXB | Glycopeptide | AGGCACAAAATCGAAGATGCTT | GGGTATGGCTCATCAATCAACTT |
| vanYB | Glycopeptide | GGCTAAAGCGGAAGCAGAAA | GATATCCACAGCAAGACCAAGCT |
| vanYD | Glycopeptide | AAGGCGATACCCTGACTGTCA | ATTGCCGGACGGAAGCA |
| IS1111 | Insertional | GTCTTAAGGTGGGCTGCGTG | CCCCGAATCTCATTGATCAGC |

| | | | |
|---|---|---|---|
| IS1133 | Insertional | GCAGCGTCGGGTTGGA | ACGCGTTCGAACAACTGTAATG |
| ISAba3-Acineto | Insertional | TCAGAGGCAGCGGTATACGA | GGTTGATTCAGTTAAAGTACGTAAAACTTT |
| ISEfm1-Entero | Insertional | AGGTGTCCATGACGTGAAAGTG | TCCTTTGTCCCCTAGGATATTGG |
| ISPps1-pseud | Insertional | CACACTGCAAAAACGCATCCT | TGTCTTTGGCGTCACAGTTCTC |
| ISSm2-Xanthob | Insertional | TGGATCGACCGGTTCCAT | GCTGACCGAGCTGTCCATGT |
| orf37-IS26 | Insertional | GCCGGGTTGTGCAAATAGAC | TGGCAATCTGTCGCTGCTG |
| orf39-IS26 | Insertional | GCGCGTCGAGCATCAATAG | CAGTTGTGCTGCTGGTGGTC |
| TN5 | Insertional | CAGCATAAAAAATCCCGACAACA | CCCCGCAACAGACATACGT |
| int1 | Integrase | GCCTTGATGTTACCCGAGAG | GATCGGTCGAATGCGTGT |
| intl2 | Integrase | TGCTTTTCCCACCCTTACC | GACGGCTACCCTCTGTTATCTC |
| intl3 | Integrase | GCCACCACTTGTTTGAGGA | GGATGTCTGTGCCTGCTTG |
| ere(A) | MLSB | GATAATTCTGCTGGCGCACA | GCAGGCGTGGTCACAAC |
| ere(B) | MLSB | TCGTATATGGCGGGCGTAGTA | GGTCCAAGATGGGTGAATGCA |
| erm(34) | MLSB | AAAGCGGTTTACAAGCGTTTCG | GGGTGCTCTAGGGTTGTTTAGTG |
| erm(35) | MLSB | CCTTCAGTCAGAACCGGCAA | GCTGATTTGACAGTTGGTGGTG |
| erm(36) | MLSB | GGCGGACCGACTTGCAT | TCTGCGTTGACGACGGTTAC |
| erm(42) | MLSB | TGTTGAGATTGGGCCTGGA | CTAAGGGTGGGTTCTCACTATCTA |
| erm(K) | MLSB | GTTTGATATTGGCATTGTCAGAGAAA | ACCATTGCCGAGTCCACTTT |
| ermA | MLSB | TCGTTGAGAAGGGATTTGCGA | TTGCATGCTTCAAAGCCTGTC |
| ermA_ermTR | MLSB | ACATTTTACCAAGGAACTTGTGGAA | GTGGCATGACATAAACCTTCATCA |
| ermB | MLSB | GAACACTAGGGTTGTTCTTGCA | CTGGAACATCTGTGGTATGGC |
| ermD | MLSB | TTTCCGGACAGCATTTGATGC | TCCACTGCCAATACCTTACCG |
| ermE | MLSB | GTCACGCAGCTGGAGTTCG | CGGTGAAGCACAGCTCGAC |
| ermF | MLSB | CAGCTTTGGTTGAACATTTACGAA | AAATTCCTAAAATCACAACCGACAA |
| ermG | MLSB | CCCTTGAATTAGTACAGAGGTG | GCAAACTCGTATTCCACGA |
| ermO | MLSB | TGATGACGGCTCAGTGG | GTGCACCAGCGCCTGA |
| ermQ | MLSB | TGAAAGCCATGCGTCTGAC | TTCAGCTGGCAGCTTAAGC |
| ermS | MLSB | GAGTACGCCCGCAAACG | GCGTTCGATCCGGAGGA |
| ermT | MLSB | GTTCACTAGCACTATTTTTAATGACAGAAGT | GAAGGGTGTCTTTTTAATACAATTAACGA |
| ermX | MLSB | GCTCAGTGGTCCCCATGGT | ATCCCCCCGTCAACGTTT |
| ermY | MLSB | TTGTCTTTGAAAGTGAAGCAACAGT | TAACGCTAGAGAACGATTTGTATTGAG |
| lmrA | MLSB | TTCAGATGCAATGGCGTTTG | ATAATCGGGAACATAATGAGCATAACTAC |
| lnu(F) | MLSB | ATACCGGTCATTTCCACTTGGC | GCATCAGGCTGATGAGGTTCAA |
| lnuA | MLSB | TGACGCTCAACACACTCAAAAA | TTCATGCTTAAGTTCCATACGTGAA |
| lnuB | MLSB | GGATCGTTTACCAAAGGAGAAGG | AGCATAGCCTTCGTATCAGGAA |
| lnuC | MLSB | GGGTGTAGATGCTCTTCTTGGA | CTTTACCCGAAAGAGTTTCTACCG |
| mef(B) | MLSB | CCGATAGGCTTACTTGTTGCAG | AGTCCACTTGCGGTTTCATTG |
| mefA | MLSB | TAATTATCGCAGCAGCTGGTTC | GTTCCCAAACGGAGTATAAGAGTG |
| mphA | MLSB | TCAGCGGGATGATCGACTG | GAGGGCGTAGAGGGCGTA |
| mphB | MLSB | CGCAGCGCTTGATCTTGTAG | TTACTGCATCCATACGCTGCTT |
| oleC | MLSB | CCCGGAGTCGATGTTCGA | GCCGAAGACGTACACGAACAG |
| pica | MLSB | GCAATCGAGGCGGTGTTC | TTGCCGCAGCCAATTCA |
| pikR2 | MLSB | TCGTGGGCCAGGTGAAGA | TTCCCCTTGCCGGTGAA |
| vat(A) | MLSB | ATGAACGGAGCGAATCATCGG | CCATACCGATCCAAACGTCATTTC |
| vat(E) | MLSB | GACCGTCCTACCAGGCGTAA | TTGGATTGCCACCGACAATT |
| vatB | MLSB | GCAATTGTTGCTGCGAATTCAG | GTGCTGACCAATCCCACCA |
| vga(A)LC | MLSB | GTGAAGATGTCTCGGGTACAATTG | GAAATACCAGGATTCCCATGCAC |
| acrA | Multidrug | GGTCTATCACCCTACGCGCTATC | GCGCGCACGAACATACC |
| acrB | Multidrug | AGTCGGTGTTCGCCGTTAAC | CAAGGAAACGAACGCAATACC |
| acrF | Multidrug | GCGGCCAGGCACAAAA | TACGCTCTTCCCACGGTTTC |
| acrR | Multidrug | GCGCTGGAGACACGACAAC | GCCTTGCTGCGAGAACAAA |
| adeA | Multidrug | CAGTTCGAGCGCCTATTTCTG | CGCCCTGACCGACCAAT |
| adeI | Multidrug | CAGTCTGGTTTGCAGTAACCA | CACTCCTACAACAACAGGCAA |
| arsA | Multidrug | CAGGTCAGCCGCATCAACC | GCCTGAAACACGGCAATTTCTTC |
| bexA_norM | Multidrug | TCGGGCATCCCGTTTATGATC | GTAGGCTGCGCATAATACCCA |
| cadC | Multidrug | CGCTCTGTGTCAGGATGAAGAG | CTTTCTTATGTGCTAGGGCGATCA |
| cefa_qacelta | Multidrug | TAGTTGGCGAAGTAATCGCAAC | TGCGATGCCATAACCGATTATG |
| ceoA | Multidrug | ATCAACACGGACCAGGACAAG | GGAAAGTCCGCTCACGATGA |
| cfr | Multidrug | GCAAAATTCAGAGCAAGTTACGAA | AAAATGACTCCCAACCTGCTTTAT |
| cmr | Multidrug | CGGCATCGTCAGTGGAATT | CGGTTCCGAAAAAGATGGAA |
| cmx | Multidrug | GCGATCGCCATCCTCTGT | TCGACACGGAGCCTTGGT |
| copA | Multidrug | TGCACCTGACVGGSCAYAT | GVACTTCRCGGAACATRCC |
| emrB_qacA | Multidrug | CTTTTCTCTAACCGTACATTATCTACGATAAA | AGAACGTAGCGACTGATAAAATGCT |
| emrD | Multidrug | CTCAGCAGTATGGTGGTAAGCATT | ACCAGGCGCCGAAGAAC |
| fexA | Multidrug | TGGTGTGGCTGTTGCAATCTTA | CCAAGGTACAAAGCACCTTGGA |
| floR | Multidrug | AACCCGCCCTCTGGATCA | GCCGTCGAGAAGAAGACGAA |
| lsaC | Multidrug | AAACGGCGTGAAAGTATCAGG | TTGTGGTGATGTAACGGATGC |
| marR | Multidrug | GCTGTTGATGACATTGCTCACA | CGGCGTACTGGTGAAGCTAAC |
| mdtA | Multidrug | ACAAGCCCAGGGCCAAC | CCTTAATGGTGCCTTCGGTTTC |
| mdtE/yhiU | Multidrug | CGTCGGCGCACTCGTT | TCCAGACGTTGTACGGTAACCA |
| mdtG | Multidrug | TTCCAGCCGGTCAGCAA | GACATCTCCCGCGAGTTCG |
| mdtH | Multidrug | ATGCTGGCTGTACAAGTGATG | CACTCCAGCGGGCGATA |
| mepA | Multidrug | ATCGGTCGCTCTTCGTTCAC | ATAAATAGGATCGAGCTGCTGGAT |
| merA | Multidrug | GTGCCGTCCAAGATCATG | GGTGGAAGTCCAGTAGGGTGA |
| mexA | Multidrug | AGGACAACGCTATGCAACGAA | CCGGAAAGGGCCGAAAT |
| mexB | Multidrug | CTGGAGATCGACGACGAGAAG | GAAATCGTTGACGTAGCTGGAA |
| mexE | Multidrug | GGTCAGCACCGACAAGGTCTAC | AGCTCGACGTACTTGAGGAACAC |

| | | | |
|---|---|---|---|
| msr(A) | Multidrug | CTGCTAACACAAGTACGATTCCAAAT | TCAAGTAAAGTTGTCTTACCTACACCATT |
| msr(C) | Multidrug | TCAGACCGGATCGGTTGTC | CCTATTTTTTGGAGTCTTCTCTCTAATGTT |
| msr(D) | Multidrug | GGCAAGCTAGGTGTTGAGC | ATTGCTCAACACCTAGCTTGC |
| msr(E) | Multidrug | CGGCAGATGGTCTGAGCTTAAA | CGCACTCTTCCTGCATAAAGGA |
| mtrD | Multidrug | CGGAGTCCATCGACCATTTG | ATCGTCGGCAAGGAGAATCA |
| mtrE | Multidrug | CGATGTGTCGTTTTGGAAGGT | CCTGCACCATGATTCCTCAATA |
| multidrug resistance | Multidrug | AATTTTGCCGATTATTGCTGAAA | GATTGTCATCATTCGTTTATCACCAA |
| nimE | Multidrug | TGCGCCAAGATAGGGCATA | GTCGTGAATTCGGCAGGTTTA |
| norA | Multidrug | ATCGCCGTTTGGTGGTACG | TCCACCAATCCCTGGTCCTAAA |
| oprD | Multidrug | ATGAAGTGGAGCGCCATTG | GGCCACGGCGAACTGA |
| optrA | Multidrug | GGTGGATGAAGTCCGTACGG | AGGTTAGACCTCCAAGAGCCA |
| pbrT | Multidrug | GATGCGCACTGGGCTTG | TCGGAATATGCGGAAATGCG |
| pcoA | Multidrug | TGGCGTATGGAGTTTCAATGC | GAATAATGCCGTGCCAGTGAA |
| qacA_B | Multidrug | AAGGGCCACTGCATTAGCTG | CCAGTCCAATCATGCCTGCA |
| qacF_H | Multidrug | TCGCAACATCCGCATTAAAA | ATGGATTTCAGAACCAGAGAAAGAAA |
| qacH_351 | Multidrug | GTCGGTGTTGCTTATGCAGTCT | CAACCAGGCAATGGCTGTAA |
| qepA_1_2 | Multidrug | GGGCATCGCGCTGTTC | GCGCATCGGTGAAGCC |
| silE | Multidrug | GGTGGAAAGTCATCAGAGGATGA | CAAAGCCCAGCAAGGATGC |
| sugE | Multidrug | CTTAGTTATTGCTGGTCTGCTGGA | GCATCGGGTTAGCGGACTC |
| tcrB | Multidrug | GTGCCGGAACTCAAGTAGCA | GCACCGACTGCTGGACTTAA |
| terW | Multidrug | TCAAAGAGCTACGCGAGTCATA | CCTTCCCTGTGGACTCACC |
| tolC | Multidrug | GGCCGAGAACCTGATGCA | AGACTTACGCAATTCCGGGTTA |
| ttgA | Multidrug | ACGCCAATGCCAAACGATT | GTCACGGCGCAGCTTGA |
| ttgB | Multidrug | TCGCCCTGGATGTACACCTT | ACCATTGCCGACATCAACAAC |
| vgaA | Multidrug | GGAAGCTATAGAGGCGTTTGAATC | CCGAAGGTTCAATACTCAATCGAC |
| vgaB | Multidrug | TAAAAGAGAATAAGGCGCAAGGA | TGTTTAGTAGCATGTTGCATTTTCC |
| bacA | other | ATCCGCGGCACCCTGA | CCTGCTTGATGGACTTGATGAAGA |
| fabK | other | CAGGAGCAGGAAATCCAAGC | CCAGCTTCCATTCCTTCTGC |
| folA | other | CGAGCAGTTCCTGCCAAAG | CCCAGTCATCCGGTTCATAATC |
| fosb | other | CTTGCAGGCCTATGGATTGC | TCTGTTCTCAAGTGTGCCAGTA |
| fosX | other | AGCTGGTTTGTGGATTTGCA | CCACACCGAGAGCTTTAATCCG |
| mcr-1 | other | CACATCGACGGCGTATTCTG | CAACGAGCATACCGACATCG |
| mcr-2 | other | CGGCGTACTTTAAGCGTTATGATG | GCATTTGGCATACCATGCAGATAG |
| nisB | other | GGGAGAGTTGCCGATGTTGTA | AGCCACTCGTTAAAGGGCAAT |
| sat-4 | other | GAATGGGCAAAGCATAAAAACTTG | CCGATTTTGAAACCACAATTATGATA |
| cat | Phenicol | ATCGGCCAGACTGGATATCGA | CACAGCTCCAGTTGCAACAAC |
| cat(pC221) | Phenicol | AATGACCGTATGCTGCAAGAAG | TTTGCCTGCTATGGCATTCTG |
| catB2 | Phenicol | GCTACTATTCCGGCTATTACCATG | GGGCTCCTCGTTCATGTAGA |
| catB3 | Phenicol | GCACTCGATGCCTTCCAAAA | AGAGCCGATCCAAACGTCAT |
| catB8 | Phenicol | CACTCGACGCCTTCCAAAG | CCGAGCCTATCCAGACATCATT |
| catB9 | Phenicol | CACCTTATGAAGTGGTCGGTTCA | GTCTGATGAACACAGAGACTGCA |
| catI | Phenicol | GGGTGAGTTTCACCAGTTTTGATT | CACCTTGTCGCCTTGCGTATA |
| catII | Phenicol | CCTGGAACCGCAGAGAACA | CGGAACTCCGGAAACTGATTAAC |
| catIII | Phenicol | CTGATTGCTCAGGCCGTGAA | ATGAGTATGGGCAACTCAGTGC |
| catP | Phenicol | CCTTTGGACTGAGTGTAAGTCTGA | TAAAGCCATCGAAGGTTGACCA |
| catQ | Phenicol | AGGTGCACTTACAGTATGACTGC | AACGTGGGAAGTTCTCGTCATAC |
| cmlA1 | Phenicol | TAGGAAGCATCGGAACGTTGAT | CAGACCGAGCACGACTGTTG |
| cmlA5 | Phenicol | GCGCTCTTCGAGGATTCG | CCGCCCAAGCAGAAGTAGAC |
| cmlV | Phenicol | GCCCTCATCACCGTCTTCG | GGACGTTGGCGATGGAGAG |
| IncN_oriT | plasmid | TTGGGCTTCATAGTACCC | GTGTGATAGCGTGATTTATGC |
| IncN_rep | plasmid | AGTTCACCACCTACTCGCTCCG | CAAGTTCTTCTGTTGGGATTCCG |
| IncP_oriT | plasmid | CAGCCTCGCAGAGCAGGAT | CAGCCGGGCAGGATAGGTGAAGT |
| IncQ_oriT | plasmid | TTCGCGCTCGTTGTTCTTCGAGC | GCCGTTAGGCCAGTTTCTCG |
| IncW_trwAB | plasmid | AGCGTATGAAGCCCGTGAAGGG | AAAGATAAGCGGCAGGACAATAACG |
| pAKD1-IncP-1β | plasmid | GGTAAGATTACCGATAAACT | GTTCGTGAAGAAGATGTA |
| PAMBL-1-F | plasmid | CAGGCTCTTAATGTGATA | TTATGCTCAATACTCGTG |
| pBS228-IncP-1α | plasmid | CAATCCATCGACAATCAC | GACAATCAGCTACTTCAC |
| tra-A | plasmid | AAGTGTTCAGGGTGCTTCTGCGC | GTCATGTACATGATGACCAAAA |
| traN | plasmid | GCTTGGCGGTCAGCAATT | TTAGGAATAACAATCGCTACACCTTTA |
| trb-C | plasmid | CGGYATWCCGSCSACRCTGCG | GCCACCTGYSBGCAGTCMCC |
| arr-2 | rifamycin | TTGGCGATTGGTGACTTGCTAA | ATCGTCTTCGAACGGTCCTG |
| arr-3 | rifamycin | GATCGTCTTCGAACGGTCCTG | TTTGGCGATTGGTGACTTGCT |
| sul1 NEW | Sulfonamide | GCCGATGAGATCAGACGTATTG | CGCATAGCGCTGGGTTTC |
| sul2 | Sulfonamide | TCATCTGCCAAACTCGTCGTTA | GTCAAAGAACGCCGCAATGT |
| sul3 | Sulfonamide | CGCGCTCAAGGCAGATG | GGGAATGCCATCTGCCTTG |
| sulA_folP | Sulfonamide | CAGGCTCGTAAATTGATAGCAGAAG | CTTTCCTTGCGAATCGCTTT |
| tet(36) | Tetracycline | AGAATACTCAGCAGAGGTCAGTTCCT | TGGTAGGTCGATAACCCGAAAAT |
| tet(38) | Tetracycline | AAGCGACATTAGCCGGTTTAG | CTGCTCGTACTTAAGCCAAGG |
| tet(39) | Tetracycline | TATAGCGGGTCCGGTAATAGGTG | CCATAACGATCCTGCCCATAGATAAC |
| tet(40) | Tetracycline | CTGTCCGTGCGCAATATATCC | GGATATATTGCGCACGGACAG |
| tet(44) | Tetracycline | CTCATGTAGATGCAGGAAAGACG | GTAACTGCTGCCTGAATTGTGA |
| tet32 | Tetracycline | CCATTACTTCGGACAACGGTAGA | CAATCTCTGTGAGGGCATTTAACA |
| tetA | Tetracycline | CTCACCAGCCTGACCTCGAT | CACGTTGTTATAGAAGCCGCATAG |
| tetA(P) | Tetracycline | GGAAACCTTAGTTCAGTGACTTGG | CCCATTTAACCACGCACTGAA |
| tetB | Tetracycline | AGTGCGCTTTGGATGCTGTA | AGCCCCAGTAGCTCCTGTGA |
| tetB(P) | Tetracycline | TGGGCGACAGTAGGCTTAGAA | TGACCCTACTGAAACATTAGAAATATACCT |
| tetC | Tetracycline | ACTGGTAAGGTAAACGCCATTGTC | ATGCATAAACCAGCCATTGAGTAAG |

| | | | |
|---|---|---|---|
| tetD | Tetracycline | AATTGCACTGCCTGCATTGC{EndPos:952} | GACAGATTGCCAGCAGCAGA{EndPos:1127} |
| tetE | Tetracycline | TTGGCGCTGTATGCAATGAT | CGACGACCTATGCGATCTGA |
| tetG | Tetracycline | TCGCGTTCCTGCTTGCC | CCGCGAGCGACAAACCA |
| tetH | Tetracycline | TTTGGGTCATCTTACCAGCATTAA | TTGCGCATTATCATCGACAGA |
| tetJ | Tetracycline | CAGCGCCCATACGCCATTTA | CCTACTTCAGTAGTGTGCCAAGC |
| tetK | Tetracycline | CAGCAGTCATTGGAAAATTATCTGATTATA | CCTTGTACTAACCTACCAAAAATCAAAATA |
| tetL | Tetracycline | ATGGTTGTAGTTGCGCGCTATAT | ATCGCTGGACCGACTCCTT |
| tetM | Tetracycline | GGAGCGATTACAGAATTAGGAAGC | TCCATATGTCCTGGCGTGTC |
| tetO | Tetracycline | CAACATTAACGGAAAGTTTATTGTATACCA | TTGACGCTCCAAATTCATTGTATC |
| tetPB | Tetracycline | TGGCAAGACGAGTTTGACTGA | GATCGCTCCACTTCAGCGATAA |
| tetQ | Tetracycline | CGCCTCAGAAGTAAGTTCATACACTAAG | TCGTTCATGCGGATATTATCAGAAT |
| tetR | Tetracycline | CCGTCAATGCGCTGATGAC | GCCAATCCATCGACAATCACC |
| tetS | Tetracycline | TTAAGGACAAACTTTCTGACGACATC | TGTCTCCCATTGTTCTGGTTCA |
| tetT | Tetracycline | CCATATAGAGGTTCCACCAAATCC | TGACCCTATTGGTAGTGGTTCTATTG |
| tetU | Tetracycline | GTGGCAAAGCAACGGATTG | TGCGGGCTTGCAAAACTATC |
| tetW | Tetracycline | ATGAACATTCCCACCGTTATCTTT | ATATCGGCGGAGAGCTTATCC |
| tetX | Tetracycline | AAATTTGTTACCGACACGGAAGTT | CATAGCTGAAAAAATCCAGGACAGTT |
| cro | Transposase | AGATGTTATCGACCACTTCGGA | CCGCTTGGCGATAAGCG |
| EAE_05855 | Transposase | CCCATCACCGCTGAACTGG | TGGGCGCTGCCATCTAAAC |
| IncHI2-smr0018 | Transposase | ATAATGATTCACCGGGGTAG | CTTCAGGCTATCGTTTCG |
| IncI1_repI1 | Transposase | CGAAAGCCGGACGGCAGAA | TCGTCGTTCCGCCAAGTTCGT |
| IncN_korA | Transposase | GGAACGTTTGTAYCTTGTATTG | ACTCACTATCTTCTGTTGATTG |
| IS1247 | Transposase | CGGCCGTCACTGACCAA | TCGGCAGGTTGGTGACG |
| IS15DI | Transposase | CAATACCTTTGATGGTGGCGTAAG | CTTACGCCACCATCAAAGGTATTG |
| IS200-1 | Transposase | CCAAATACCGAAGACAAGCGTTC | CCAAACTGCTCGTAAAGCATCAG |
| IS200-2 | Transposase | GCACACCCGATGGAACTGTAAA | TCGGCGGGATCTCCAGAAG |
| IS21-ISAs29 | Transposase | GGTCCGTCAGGCACAAGTC | GGGATCGTATCGGCAAGCC |
| IS256 | Transposase | CTTGCGCATCATTGGATGATGG | AAGAACGGCTCCAATTAAGCGA |
| IS26 | Transposase | ATGGATGAAACCTACGTGAAGGTC | CGGTACTTAATCTGTCGGTGTTCA |
| IS3 | Transposase | CGGTCTGAGCTTCGGGAA | AGAACTGTCACTCCGGTCTG |
| IS5/IS1182 | Transposase | TTCTCGAAGAATCGCCATGGC | GCTTTGGATCGCTCCAATCGA |
| IS6/257 | Transposase | ATATCGTGCCATTGATGCAGAG | ACCATTGCTACCTTCGTTGAAG |
| IS6100 | Transposase | CGCACCGGCTTGATCAGTA | CTGCCACGCTCAATACCGA |
| IS613 | Transposase | AGGTTCGGACTCAATGCAACA | TTCAGCACATACCGCCTTGAT |
| IS630 | Transposase | CCGCCACCAGTGTGATGG | TTGGCGCTGACTGGATGC |
| IS91 | Transposase | GGATGCCACTGCTGGTCA | ACAGTGGATACAGTATCTGCTGAG |
| ISCR1 | Transposase | ATGGTTTCATGCGGGTT | CTGAGGGTGTGAGCGAG |
| ISEcp1 | Transposase | CATGCTCTGCGGTCACTTC | GACGCACCTTCTTGATGACC |
| lncF_FIC | Transposase | GTGAACTGGCAGATGAGGAAGG | TTCTCCTCGTCGCCAAACTAGAT |
| mobA | Transposase | GCTTCCCGTAACGAGGTAGT | CCTTGAACGGTATCAGCACG |
| Tn3 | Transposase | GCTGAGGTGTTCAGCTACATCC | GCTGAGGTAGTCACAGGCATTC |
| TN5403 | Transposase | AAGCGAATGGCGCGAAC | CGCGCAGGGTAAACTGC |
| tnpA-1 | Transposase | GCCGCACTGTCGATTTTTATC | GCGGGATCTGCCACTTCTT |
| tnpA-2 | Transposase | CCGATCACGGAAAGCTCAAG | GGCTCGCATGACTTCGAATC |
| tnpA-3 | Transposase | GGGCGGGTCGATTGAAA | GTGGGCGGGATCTGCTT |
| tnpA-4 | Transposase | CATCATCGGACGGACAGAATT | GTCGGAGATGTGGGTGTAGAAAGT |
| tnpA-5 | Transposase | GAAACCGATGCTACAATATCCAATTT | CAGCACCGTTTGCAGTGTAAG |
| tnpA-6 | Transposase | TGCAGATGGTTTAACCTTGGATATTT | TCGGTTCATCAAACTGCTTCAC |
| tnpA-7 | Transposase | AATTGATGCGGACGGCTTAA | TCACCAAACTGTTTATGGAGTCGTT |
| Tp614 | Transposase | GGAAATCAACGGCATCCAGTT | CATCCATGCGCTTTTGTCTCT |
| trfa | Transposase | ACGAAGAAATGGTTGTCCTGTTC | CGTCAGCTTGCGGTACTTCTC |
| dfrA1 | trimethoprim | GGAATGGCCCTGATATTCCA | AGTCTTGCGTCCAACCAACAG |
| dfrA10 | trimethoprim | CTTCAACTATCACAGAGCACGAAG | TCTACCGGTACATACACATCAGC |
| dfrA12 | trimethoprim | CCTCTACCGAACCGTCACACA | GCGACAGCGTTGAAACAACTAC |
| dfra14 | trimethoprim | CGGATCATGTCATTGTTTCAGG | ATGTTAGAGGCGAAGTCTTGG |
| dfrA15 | trimethoprim | AGGCCGAAAGACTTTCGAGTC | TCACCTTCTGGCTCAATGTCG |
| dfra17 | trimethoprim | CGGGAACGGCCCTGATATTCC | CGTGTTGCGACCGCATACTTTC |
| dfrA18 | trimethoprim | GGAGCGAATCAAGGAGAAAGGAA | GCAATGCGTTGATCGGTATTCTC |
| dfra21 | trimethoprim | TTGTTTCAACGCTGTCGCA | GGTTTCGGTTGAGACAAGCTC |
| dfrA22 | trimethoprim | CAGCCGAACACGGCAAAG | CGGAGTGCGTGTACGTGA |
| dfrA25 | trimethoprim | TCAAACTGGACAGCGGCTA | GTCGATTGTCGACACATGCA |
| dfrA27 | trimethoprim | GCCGCTCAGGATCGGTA | GTCGAGATATGTAGCGTGTCG |
| dfra5 | trimethoprim | CCATGGAGTGCCAAAGGTG | CACCTTTGGCACTCCATGG |
| dfra7 | trimethoprim | GTAATCGGTAGTGGTCCTGA | ATCAGGACCACTACCGATTAC |
| dfrA8 | trimethoprim | GGTCGCACCTGCATCGTTA | AGCGCCACCAATGACGTAG |
| dfrB4 | trimethoprim | CGGTTCGCATTCCCATCAAA | CGCAGTCATGGGATAAATCTGG |
| dfrBmulti | trimethoprim | ACCAAGGCAGAAGTGAAGTCA | GGTGAGCCTCAGACTCGAC |
| dfrC | trimethoprim | GTCGCTCACGATAAACAAAGAGTC | CCCTTCATGGTGAAATGAAGCTTG |
| dfrG | trimethoprim | TCAATCGGAAGAGCCTTACCTGA | TGGGCAAATACCTCATTCCATTCC |
| dfrK | trimethoprim | TGCTGCGATGGATAAGAACAG | CTTCCAGGTAATGCTCTTCCG |
| 16S rRNA | Bacterial marke | GGGTTGCGCTCGTTGC | ATGGYTGTCGTCAGCTCGTG |

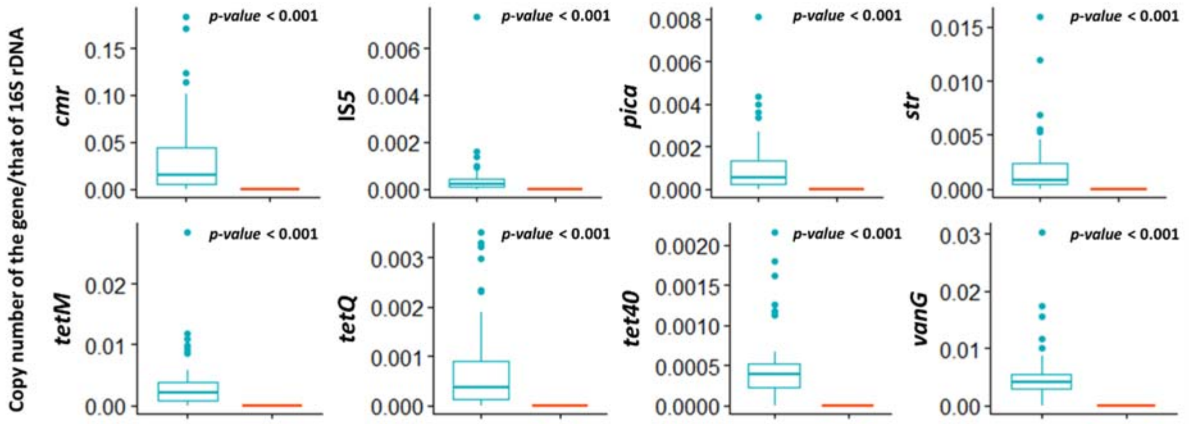


**Fig. S1. Comparison of relative abundance (target gene copies/16S rRNA gene copies) of candidate ARGs and MGEs between greenhouse (n = 41) and control (n = 19) groups.** Blue and red colors indicate greenhouse and control samples, respectively. Significance level (*p*-value) is shown in each plot. The y-axis indicates the relative concentration of each core ARG and MGE. Kruskal-Wallis test was performed to calculate the statistical significance between greenhouse and control groups.
